# Beyond Cell Motility: The Expanding Roles of Chemokines and Their Receptors in Malignancy

**DOI:** 10.3389/fimmu.2020.00952

**Published:** 2020-06-04

**Authors:** Dina Morein, Nofar Erlichman, Adit Ben-Baruch

**Affiliations:** School of Molecular Cell Biology and Biotechnology, George S. Wise Faculty of Life Sciences, Tel Aviv University, Tel Aviv, Israel

**Keywords:** atypical chemokine activities in cancer, atypical chemokine receptors, breast cancer, chemokines, classical chemokine receptors

## Abstract

The anti-tumor activities of some members of the chemokine family are often overcome by the functions of many chemokines that are strongly and causatively linked with increased tumor progression. Being key leukocyte attractants, chemokines promote the presence of inflammatory pro-tumor myeloid cells and immune-suppressive cells in tumors and metastases. In parallel, chemokines elevate additional pro-cancerous processes that depend on cell motility: endothelial cell migration (angiogenesis), recruitment of mesenchymal stem cells (MSCs) and site-specific metastasis. However, the array of chemokine activities in cancer expands beyond such “typical” migration-related processes and includes chemokine-induced/mediated atypical functions that do not activate directly motility processes; these non-conventional chemokine functions provide the tumor cells with new sets of detrimental tools. Within this scope, this review article addresses the roles of chemokines and their receptors at atypical levels that are exerted on the cancer cell themselves: promoting tumor cell proliferation and survival; controlling tumor cell senescence; enriching tumors with cancer stem cells; inducing metastasis-related functions such as epithelial-to-mesenchymal transition (EMT) and elevated expression of matrix metalloproteinases (MMPs); and promoting resistance to chemotherapy and to endocrine therapy. The review also describes atypical effects of chemokines at the tumor microenvironment: their ability to up-regulate/stabilize the expression of inhibitory immune checkpoints and to reduce the efficacy of their blockade; to induce bone remodeling and elevate osteoclastogenesis/bone resorption; and to mediate tumor-stromal interactions that promote cancer progression. To illustrate this expanding array of atypical chemokine activities at the cancer setting, the review focuses on major metastasis-promoting inflammatory chemokines—including CXCL8 (IL-8), CCL2 (MCP-1), and CCL5 (RANTES)—and their receptors. In addition, non-conventional activities of CXCL12 which is a key regulator of tumor progression, and its CXCR4 receptor are described, alongside with the other CXCL12-binding receptor CXCR7 (RDC1). CXCR7, a member of the subgroup of atypical chemokine receptors (ACKRs) known also as ACKR3, opens the gate for discussion of atypical activities of additional ACKRs in cancer: ACKR1 (DARC, Duffy), ACKR2 (D6), and ACKR4 (CCRL1). The mechanisms involved in chemokine activities and the signals delivered by their receptors are described, and the clinical implications of these findings are discussed.

## Introduction

Leukocyte trafficking is the hallmark of immune integrity, directing the appropriate positioning of lymphocytes and myeloid cells in tissues during acquired immunity, inflammation, and immune homeostasis. These processes are controlled by a very large array of chemotactic molecules—chemokines and others—that act in an orchestrated manner to achieve accuracy, fine-tuning, and precise turn-on/turn-off signals in regulating leukocyte influxes (1–3).

In addition, chemotactic cues that are largely mediated by chemokines and their receptors are strongly involved in the dynamic processes of tumor development and progression. In line with their key roles in regulating leukocyte trafficking under physiological conditions, chemokines and their G protein-coupled receptors (GPCRs) are central players in dictating the types and amounts of leukocytes that are recruited to tumors and metastases (4–7). For example, at relatively early stages of the malignancy process, chemokines can induce the infiltration of lymphocytes that have the potential to raise anti-tumor activities. This is illustrated by Th1 cells, cytotoxic T cells (CTLs) and natural killer cells (NK). However, gradually, the leukocyte contexture at the tumor site is changed in chemokine-driven manner toward an immune-suppressive and pro-inflammatory type, where chronic inflammation turns into a deleterious force that was termed “The Seventh Hallmark of Cancer.” Here, the cellular infiltrates can include inflammatory macrophages that are typically regarded as M1 macrophages, as well as M2 macrophages that constitute an important essence of tumor-associated macrophages (TAMs); they can also include neutrophils that are sub-divided to N1 and N2 types and myeloid-derived suppressor cells (MDSCs) of the monocytic (M-MDSCs) or granulocytic (G-MDSCs) subsets. In parallel, T regulatory cells (Tregs) can put their marks on the process, usually contributing to immune suppression (but in other cases they can also be beneficial by inhibiting chronic inflammation) (4–12).

With time, it was realized that other processes that depend on chemokine-induced cell motility can also take place in the tumor context. Well-known are the functions of chemokines in regulating the migration of endothelial cells and their progenitors during angiogenesis; these processes are typically induced by ELR+ CXC chemokines, by CXCL12 and by some of the CC chemokines, but can alternatively be inhibited by non-ELR CXC chemokines. Chemokines also regulate the migration of mesenchymal stem cells (MSCs) to tumor sites, where they can express a variety of pro-cancerous activities and differentiate to tumor-promoting cancer-associated fibroblasts (CAFs). Moreover, chemokines expressed in metastatic sites are key players in attracting to these organs tumor cells that express the corresponding receptors. This venue has been predominantly demonstrated by the CXCL12-CXCR4 axis but also by other chemokine-chemokine receptor pairs, mostly of the homeostatic sub-family. All of these aspects of chemokine activities in cancer have been broadly reviewed, and representative summarizing articles covering these different aspects are provided (4, 5, 7, 13–26).

At this point in time, research on chemokine activities in cancer—that are not directly mediated by cell migration, e.g., in response to chemotactic gradients—is rapidly growing, providing insights to atypical activities of different members of the family in many cancer types. In this review, we describe such non-conventional chemokine activities in cancer, exerted directly on the tumor cells and at the tumor microenvironment (TME). As will be described below, chemokines can promote cancer cell proliferation and survival, reduce their apoptosis and control their senescence; chemokines can also enrich the sub-population of cancer stem cells (CSCs) in tumors, facilitate tumor cell spreading by promoting epithelial-to-mesenchymal transition (EMT) and the release of matrix metalloproteinases (MMPs) in the cancer cells, and increase tumor cell resistance to therapy. In parallel, atypical chemokine-mediated effects can promote interactions between cancer cells and their microenvironment in a way that can also contribute to tumor progression: chemokine activities reduce the efficacy of immune checkpoint blockades (ICBs), induce bone remodeling processes that support the metastatic cascade and enhance the tumor-promoting interactions of cancer cells with stromal cells, such as MSCs and CAFs.

To exemplify the atypical activities of chemokines in cancer, we focus in this review on the effects of inflammatory chemokines that play causative tumor-promoting roles in many malignancies, and whose migration-related functions in cancer have been comprehensively described in many review articles [representative review articles are given as references (4, 5, 7, 13–26)]. In this context, emphasis is put mainly on ELR+ CXC chemokines that act through CXCR1/CXCR2 (e.g., CXCL1, CXCL5, CXCL8), CCL2 that signals mainly *via* the CCR2 receptor and CCL5 with its CCR5 receptor. In parallel, the review also addresses CXCL12—that can exert inflammatory and homeostatic activities—and its CXCR4 receptor, due to their major involvement at all stages of tumor progression. The major findings described herein are summarized in Table 1.

**Table 1:** Atypical chemokine functions in cancer, mediated by axes of chemokines and classical chemokine receptors.

**Axis**	ATYPICAL tumor-related activities induced *via* CLASSICAL chemokine receptors^*^	**Effect**
**CXCR1/CXCR2** CXCL1 CXCL5 CXCL8	• Increases tumor cell proliferation, viability and anchorage independent cell growth • Reduces cancer cell apoptosis • Down-regulates tumor senescence; Increases senescence, which is accompanied by elevated pro-metastatic potential • Enriches the CSC sub-population • Elevates EMT properties and tumor cell invasion • Increases MMP production by cancer cells • Promotes chemoresistance and endocrine resistance of tumor cells • Elevates the expression of inhibitory immune checkpoints (PD-L1) by cancer cells and immune cells • Reduces the efficacy of immunotherapy • Promotes osteoclastogenesis and bone damage • Drives forward pro-cancerous tumor-stroma interactions	Pro-cancerous
**CCR2** CCL2	• Increases breast tumor proliferation and survival • Reduces cancer cell apoptosis • Elevates tumor cell invasion (including *via* CCL2 that is released by senescent tumor cells) • Enriches the CSC sub-population • Elevates EMT properties and tumor cell invasion • Promotes endocrine resistance of tumor cells • Reduces the efficacy of immunotherapy • Promotes osteoclast differentiation and bone resorption • Drives forward pro-cancerous tumor-stroma interactions	Pro-cancerous
**CCR5** CCL5	• Increases tumor cell proliferation (particularly in the context of hormonal stimulation) • Elevates tumor cell invasion (including *via* CCL5 that is released by senescent fibroblasts) • Enriches the CSC sub-population • Elevates EMT properties and tumor cell invasion • Elevates the expression of inhibitory immune checkpoints (PD-L1) by cancer cells • Reduces the efficacy of immunotherapy • Drives forward pro-cancerous tumor-stroma interactions ------------------------------------------------------------------ • Inhibits tumor cell proliferation • Promotes the efficacy of ICBs (*via* recruitment of T effector cells)	Mostly pro-cancerous
**CXCR4** CXCL12	• Increases tumor cell proliferation • Induces EGFR transactivation in cancer cells • Elevates collective invasion and elevates survival of non-senescent cells (*via* CXCL12 released by senescent tumor cells) • Enriches the CSC sub-population • Elevates EMT properties and tumor cell invasion • Increases MMP production by cancer cells • Promotes endocrine resistance of tumor cells • Elevates the expression of inhibitory immune checkpoints (PD-L1) by cancer cells • Reduces the efficacy of immunotherapy • Promotes (together with TGFβ) fibroblast transition to CAFs and drives forward pro-cancerous tumor-stroma interactions	Pro-cancerous

*The Table summarizes the effects of axes established between chemokines and their classical receptors (that signal via heterotrimeric G proteins) on atypical cancer-related activities (that are not directly mediated by cell motility)*.

* *Most of these findings were obtained in breast cancer studies, as described in the text. CAFs, Cancer-associated fibroblasts; CSC, Cancer stem cells; EGFR, Epithelial growth factor receptor; EMT, Epithelial-to-mesenchymal transition; ICBs, Immune checkpoint blockades; MMPs, Matrix metalloproteinases; TGF, Transforming growth factor. The dashed line separates the pro-malignancy activities of CCL5, which mostly dominate its effects in cancer (above the line), from its anti-malignancy roles (below the line)*.

In the context of CXCL12 activities in cancer, the review also addresses the roles of CXCR7 which is the other CXCL12 receptor; here, we describe the functions of CXCR7 alone or in the context of CXCR4, in regulating non-conventional cancer-related effects. Although these two receptors can cooperate in mediating tumor-promoting effects, anti-tumor effects of CXCR7 were reported as well, possibly resulting from its being an atypical chemokine receptor (ACKR). Like CXCR7—known also as ACKR3—other ACKRs do not transmit intracellular signals through heterotrimeric G proteins, and regulate many aspects of tumor progression (2, 4, 27). Thus, to broaden the scope of atypical activities of chemokine receptors in cancer, a section of the review is dedicated to atypical roles of additional members of the ACKRs sub-group in malignancy: ACKR1, ACKR2, and ACKR4. A summary of the key findings that are described below on ACKRs in cancer is provided in Table 2.

**Table 2:** Tumor-related activities, mediated by atypical chemokine receptors.

**Receptor**	Tumor-related activities induced *via* ATYPICAL chemokine receptors*	**Effect/s**
**ACKR1** (DARC, Duffy)	• Inhibits tumor cell proliferation and increases tumor cell senescence • Interferes with CXCR2-induced STAT3 activation in cancer cells • Reduces MMP production by tumor cells • Leads to reduced microvessel density • Single nucleotide polymorphisms affect angiogenesis, tumorigenesis and lung metastasis	Anti-cancerous;
**ACKR2** (D6, CCBP2)	• Inhibits tumor cell proliferation • Reduces cancer cell invasion • Reduces the infiltration/activities of tumor-supporting leukocytes (in parallel to lower chemokine levels) • Restricts angiogenesis ------------------------------------------------------------ • Elevates EMT properties and tumor cell migration • Prevents anti-tumor activities of NK cells and neutrophils	Anti-cancerousAt times pro-cancerous
**ACKR3** (CXCR7)	• Increases tumor cell proliferation, and reduces trail-mediated apoptosis • Induces EGFR activation • Enriches the CSC sub-population • Increases ERα stability and confers insensitivity to endocrine therapy • Leads to increased endothelial cell migration (angiogenesis) ------------------------------------------------------------------- • Inhibits cell proliferation, possibly through CXCL12 sequestration • Antagonizes the ability of CXCR4-expressing tumor cells to degrade matrix	Mostly pro-cancerous; Anti-cancerous under certain settings
**ACKR4** (CCRL1, CCX-CKR)	• Inhibits tumor cell proliferation • Reduces EMT properties and tumor cell migration • Sequesters CC chemokines in tumor xenografts ----------------------------------------------------------- • Increases resistance to anoikis • Elevates EMT in tumor cells and modifies tumor cell adhesion (cell-to-cell and to ECM)	Mostly anti-cancerous

*The Table summarizes the effects of atypical chemokine receptors (ACKRs) in breast cancer as well as in other malignancies; the findings refer to non-conventional functions (not motility-related) and to other ACKR activities as well. ECM, Extracellular matrix; EGFR, Epithelial growth factor receptor; EMT, Epithelial-to-mesenchymal transition; ER, estrogen receptor; MMPs, Matrix metalloproteinases. For each of the ACKR (ACKR2, ACKR3, ACKR4), the dashed line separates the functions that dominate its effects in cancer (above the line) from its opposing roles (below the line)*.

Of the different malignant diseases, breast cancer has been the subject of intensive research that has addressed the way chemokines affect disease progression. Thus, we hereby use breast malignancy to exemplify the non-conventional effects of the above chemokines in the cancer setting. The different published studies on chemokine roles in breast cancer addressed so far primarily two subtypes of disease: (1) The highly aggressive triple-negative (TNBC) subtype in which the tumors are negative for the expression of hormone receptors and lack HER2 amplification; these tumors commonly develop resistance to chemotherapy; (2) The luminal-A subtype in which the tumors express estrogen/progesterone receptors (but not amplified HER2) and are hormone-responsive; this disease subtype is treated by endocrine therapies and is considered as having the best prognosis out of all breast cancer subtypes (28, 29). Of note, some of the aspects are relatively newly investigated, thus not much information is available in breast cancer; in these cases the scope is expanded to other cancer types as well. Together, the findings presented in this review address the multifaceted impact that chemokines may have in cancer, through functions that are beyond the typical motility-mediated levels described so far.

## Atypical Chemokine Activities Exerted on Cancer Cells

### Tumor Cell Growth, Survival and Senescence

One of the first indications that chemokines can regulate tumor progression by acting directly on the tumor cells came from early studies in melanoma, where ELR+ CXC chemokines were found to up-regulate tumor cell proliferation. By inhibiting the expression or activities of the chemokines, the different investigations indicated that CXCL1 (MGSA) and CXCL8 up-regulated the proliferation of different melanoma cells (30–33).

Along these lines, CXCL1 as well as CXCL8 have been found to promote the proliferation of breast cancer cells. These two chemokines share high affinity binding to CXCR2, but differ in their ability to activate the CXCR1 receptor; accordingly, in some of the studies inhibitors of both receptors or only of CXCR2 (e.g., repertaxin and SB225002, respectively) were used in order to determine the involvement of these two receptors in mediating such chemokine activities. In parallel, other inhibitory measures were used in order to down-regulate the chemokine/s or their receptors, and the opposite approach of over-expression was also used to determine the roles of these chemokine axes in breast cancer progression. Together, these publications indicated that ELR+ CXC chemokines—derived from autocrine or paracrine sources—induced signaling through CXCR1/CXCR2, leading to increased tumor cell proliferation, viability and anchorage independent cell growth; the chemokines also reduced the levels of tumor cell apoptosis, and inhibition of these chemokine pathways caused cell cycle arrest. In some of the studies, the chemokines were not potent in regulating such growth-related parameters when they acted alone but they have intensified the impacts of other regulators of cell growth, such as IL-6 and chemotherapy (34–40).

In essence, similar growth-stimulating regulatory modes were also reported for the inflammatory CC chemokines CCL2 and CCL5. Here, interesting connections were found between CCL2-CCR2 and estrogen responsiveness and activities: CCL2 activated estrogen receptor α (ERα) through PI3K/Akt/mTOR signaling to elevate breast tumor cell division (41); another facet of CCL2-estrogen interactions was revealed when stimulation of luminal-A breast tumor cells by estrogen has led *via* twist activation to elevated production of CCL2, then giving rise to increased proliferation of the cancer cells (42). Another study found that CCL2 binding to CCR2 has led through MEK and ERK activation to increased cancer cell survival, partly through activation of the Rho pathway (43). In parallel, CCL2 has elevated the levels of PCNA+ cancer cells and has also shifted the cell cycle from G2-M to G1-S in association with SRC and PKC activation in TNBC cells (44). The effects of the CCL2-CCR2 axis were noted not only on breast tumor cells of different subtypes (e.g., TNBC and luminal-A) but also in mammary intra-ductal injection models that mimicked the ductal carcinoma *in situ* (DCIS) stage of disease. In this system, CCL2 provided by fibroblasts has activated CCR2 that was expressed by transformed breast cells, leading to their increased proliferation and reduced apoptosis. The opposite result was obtained when CCR2 was down-regulated in the malignant cells. These changes were noted in cells within DCIS lesions, accompanied by reduced lesion size when CCR2 expression was reduced (45).

Parallel studies on CCL5 demonstrated its ability to induce small increases in breast tumor cell proliferation; in one of the research systems, such CCL5 activity was mediated by CCR5-dependent mTOR activation (46–48). CCR5, a major CCL5 receptor, was targeted in several studies by maraviroc, leading to controversial results in terms of tumor cell proliferation (48–51), which possibly reflect the use of different model systems and/or the ability of CCL5 to activate CCR1 and CCR3 in addition to CCR5. Cooperativity between CCR5-related pathways and other elements was revealed when maraviroc—that did not act alone to prevent tumor cell survival—potentiated the effect of IL-6-directed inhibition in reducing tumor cell proliferation. Of interest is the fact that in contrast to these culture experiments, maraviroc has led to significant inhibition of tumor metastasis in animal studies (49, 51), possibly reflecting the ability of CCR5 to promote breast malignancy by additional pro-tumorigenic properties, such as those that depend on cellular migration.

Increased tumor cell proliferation and growth were also found to be exerted by CXCL12 and its two receptors, CXCR4 and CXCR7/ACKR3, primarily in the context of hormonal stimulation. Studies of luminal-A breast cancer cells, that by definition are responsive to estrogen, demonstrated that the hormone induced the expression of CXCL12 and of CXCR4 in the tumor cells, leading to enhanced tumor cell growth, and also gave rise to EGFR transactivation and then to increased DNA synthesis (52–54). Along the same lines, following EGF stimulation a CXCR7/ACKR3-mediated process of EGFR activation was revealed (possibly through β-arrestin scaffold), leading to increased tumor cell proliferation (55). Additional research in this direction provided evidence to complex roles for CXCR7/ACKR3 and for its interactions with CXCR4 in regulating the proliferation and growth of breast tumor cells. On one hand, it was found that the expression of CXCR7/ACKR3 by breast tumor cells has provided growth advantages to luminal breast tumor cells (at times even when CXCR4 was not active in this respect), and reduced trail-mediated apoptosis in such cells (56, 57). Moreover, CXCR7/ACKR3-expressing cells increased the proliferation of CXCR4-expressing tumor cells (58), and silencing experiments of CXCR4 or CXCR7/ACKR3 demonstrated that each of the two receptors elevated tumor cell growth and that the joint impact of both receptors together was stronger than of each alone (59). However, another study demonstrated different roles for CXCR4 and CXCR7/ACKR3 in regulating estrogen-dependent growth of luminal breast tumor cells, where CXCR4 enhanced cancer cell growth and CXCR7/ACKR3 over-expression inhibited cell proliferation, possibly through CXCL12 sequestration (60).

A complementary subject that is related to tumor cell survival concerns the roles of chemokines in regulating cellular senescence; this process, in which cells cannot enter cell cycle and their proliferation is halted in a permanent manner, has major roles in controlling cancer progression (61, 62). Senescent cells are metabolically active and secrete many proteins, identified as senescence-associated secretory phenotype (SASP), which includes many pro-inflammatory factors, of which a predominant factor is CXCL8 (62–64).

Although chemokine-induced senescence of tumor cells may limit tumor growth, it is possible that such growth-restraining processes may be overcome by chemokine-induced pro-malignancy activities such as tumor cell growth or invasion. The dual roles of chemokines in the senescence context are nicely exemplified by a study on human pituitary tumor-transforming gene 1 (PTTG-1)-driven expression of CXCL1 and CXCL8 in breast tumor cells. In this study, it was demonstrated that activation of CXCR2 has induced senescence in luminal-A breast tumor cells and limited tumor growth and metastasis; but in parallel, the pro-metastatic potential of the cancer cells was elevated when they were co-injected with PTTG-1-over-expressing MCF-7 cells, by creating a metastasis-promoting TME (65). Whereas, this study indicated that signaling *via* CXCR2 has increased the senescence of luminal-A breast tumor cells, in another study opposite findings were found, demonstrating that CXCR2 down-regulated senescence of breast tumor cells, including of the luminal-A subtype (66). In this respect, it was found also that fibroblast-derived SASP induced EMT in non-aggressive breast tumor cells, with direct roles of CXCL8 + IL-6 in promoting tumor cell invasiveness (67). Similarly, CCL2 that was released by senescent melanoma cells increased tumor cell invasion (68) and CCL5 derived from age-senescent fibroblasts elevated the proliferation of prostate epithelial cells (69). Along these same lines, CXCL12 that was present in SASP of senescent papillary thyroid carcinoma (PTC) cells played key roles in inducing collective invasion of the cancer cells and in increasing the survival of non-senescent PTC cells, in a CXCR4-dependent manner (70).

Chemokines released by senescent cells can also impact the type of leukocytes entering the tumor site, thus dictating the effects of the immune contexture on tumor fate. For example, CCL2 produced by oncogene-induced senescent hepatocytes had the potential to induce the recruitment of immature myeloid cells that could differentiate to macrophages, which cleared senescent tumor cells; but when the cancer has been fully established, immature myeloid cells that were recruited by CCL2-mediated signals, inhibited the anti-tumor activities of NK cells and led to increased tumor growth (71). The connection between senescence, chemokines and NK cell activities was also demonstrated in a mouse model of liver carcinoma, when inducible p53 expression has increased tumor cell senescence *via* induction of CCL2, leading to recruitment of NK cells expressing anti-tumor functions (72).

### Cancer Stem Cells

Stemness is an essential trait of malignancy, whereby a small proportion of cancer stem cells (CSCs; called also tumor-initiating cells) can generate a heterogeneous tumor cell population; the CSC sub-population is often increased following treatment and therefore is considered fundamental in development of therapy resistance (73, 74). In breast cancer, CSCs are usually defined by the CD44^+^/CD24^−/low^ phenotype, and/or as being positive for the activity of the ALDH1 enzyme which is recognized by elevated proportion of an ALDEFLOUR+ cell population; often, elevated extent/size of tumor spheroids (mammospheres) is also considered a potential marker for enrichment of CSCs (74, 75).

ELR+ CXC chemokines such as CXCL1 and CXCL8, as well as their CXCR1/CXCR2 receptors, have been demonstrated to be significant factors in promoting CSC enrichment in breast cancer. In line with findings demonstrating that CXCL8 increased the ALDEFLOUR+ population and spheroid formation in breast cancer cells (76), blockade of CXCR1, *in vitro* or *in vivo* decreased the ALDEFLOUR+ population and reduced tumor growth and metastasis; this CXCR1-mediated effect on CSC viability depended on Akt activation (77). In parallel, CXCL1 arriving from TAMs was found to promote the CD44^+^/CD24^−^ sub-population and formation of tumor spheroids in human TNBC cells (78). From the mechanistic aspect, the cross-talk between chemokine receptors and the Erb-pathway may contribute to generation of CSCs in breast cancer. This possibility is exemplified by the fact that CXCR1/2 inhibition by the antagonist SCH563705 has given rise to inhibition of spheroid formation in HER2+ breast tumor cells, and inhibition of HER2-mediated signaling by lapatinib or siHER2 has led to inhibition of CXCR1/2-dependent CSC-spheroid formation (79).

With respect to clinical relevance, a recent study indicated that CXCL8 neutralizing antibodies abrogated the ability of paclitaxel and gemcitabine to elevate CSC levels in breast cancer (tumor spheroids and ALDH-expressing cells). Here, the induction of CXCL8 by chemotherapy was mediated by HIF signaling *via* ROS-dependent expression (80). Similar findings, supporting the roles of CXCL8 and its receptors in generating CSC when breast cancer cells are exposed to chemotherapy, were found when neutralizing antibodies to CXCL8 or the CXCR1/2 inhibitor reparixin inhibited the generation of CD44^+^/CD24^−^ cells, ALDH-expressing cells and spheroid formation following paclitaxel treatment. In this study, treatment of mice with reparixin decreased the number of tumor-initiating cells, which was originally increased in the tumor as a result of chemotherapy administration (81). Another study indicated that when CXCR1 was inhibited and has led to reduced generation of spheroids and their volume, paclitaxel has further augmented this effect (35).

The two inflammatory CC chemokines, CCL2 and CCL5 were also found to elevate the generation of CSCs. This was evidenced by a CCL2-promoted formation of primary and secondary tumor spheroids that contained more self-renewing CSCs (82), and by the fact that stimulation of breast tumor cells with CCL5 increased the CD44^+^/CD24^−^ sub-population (47). These CCL5-enriched CSCs expressed higher levels of the corresponding receptor CCR5 and were able to invade more than non-CSCs, ability that was abrogated by inhibition of CCR5 (47). In another study, CCR5-expressing breast cancer cells demonstrated higher potency in forming mammospheres *in vitro* and in initiating tumor formation *in vivo*, than cells not expressing the receptor (83).

Another important axis in this respect is CXCL12-CXCR4, as demonstrated in the luminal-A subtype of breast cancer. Overexpression of CXCL12 in breast cancer cells elevated the proportion of CD44^+^/CD24^−^ cells, of ALDH-expressing cells, as well as the expression of stemness markers such as Oct4, nanog and sox2 (84). Along these lines, CXCR4-expressing tumor cells demonstrated higher ability to form mammospheres than CXCR4-negative cells (85); like CXCR4, CXCR7/ACKR3 was found to play key roles in promoting the CSC sub-population, as indicated by reduced levels of CD44^+^/CD24^low^ cells, of ALDH-expressing tumor cells and of Oct4 and nanog expression following down-regulation of CXCR7/ACKR3 (56). Following co-culturing of the tumor cells with CAFs, a process that has led to increased production of CXCL12, CXCR4 inhibition has reduced the formation of spheroids that were enriched with CD44^+^/CD24^−^ cells (86). Additional findings connected chemokines with CSCs and resistance to therapy by demonstrating that CXCR4 signaling was required for the generation of cells with CSC characteristics out of tamoxifen-resistant luminal-A breast tumor cells (87).

### Metastasis-Promoting Functions: EMT and MMPs

A major paradigm in the context of chemokine-directed site-specific metastasis is that in response to chemokines that are expressed at specific organs, tumor cells that express the corresponding receptors migrate and home to these sites. Such processes were well-exemplified for the CXCL12-CXCR4 pair, as well as for other chemokine axes in a very large number of malignant diseases [summarized for example in (23, 25, 26)]. In parallel, irrespective of directing cancer cells to defined organs in the course of metastatic spread, chemokine-induced cytoskeleton re-organization and tumor cell migration/invasion were reported in many tumor systems and were strongly connected to the ability of the cancer cells to acquire a more aggressive phenotype.

Within the scope of the current review article, we wish to expand the discussion beyond such direct chemokine activities that promote tumor cell migration and invasion, and elaborate on other chemokine-induced functions that can promote cancer cell spreading and metastasis, such as EMT and MMP release. Indeed, the arena of chemokine activities was expanded toward direct abilities of chemokines to promote in the tumor cells mesenchymal properties; the mesenchymal characteristics of cells undergoing EMT include properties such as elevated expression of vimentin and of specific transcriptional repressors (such as twist, snail, slug and zeb) alongside with reduced E-cadherin expression. As mesenchymal properties generally facilitate motility, often independently of chemotactic gradient-mediated processes, in some of the studies the elevated levels of EMT were connected to increased tumor cell migration and invasion.

For example, a recent study demonstrated that CXCL1 derived from TAMs elevated EMT properties in luminal-A and TNBC breast tumor cells, in a NF-κB-mediated process that has led to activation of SOX4 (88). Another study indicated that through the activities of the transcription factor Brachyury that has led to CXCL8 up-regulation in breast tumor cells, EMT processes were increased in adjacent cancer cells. Accordingly, CXCL8 induced tumor cell invasiveness through a Brachyury-dependent process (89). With relevance to obesity-related aspects of breast cancer, CXCL8 that was induced *via* the PI3K/Akt-mediated pathway was found to mediate the EMT-inducing effects of leptin and its ability to increase tumor cell invasion (90). Similar roles for CXCL1/CXCL8 and their receptors in inducing EMT were implicated in several other publications of breast tumor cells (66, 78, 91, 92).

In parallel, CCL2 activities *via* CCR2, as well as CCL5-induced signaling were demonstrated to contribute to increased EMT and twist expression, at times accompanied by increased tumor cell invasion in breast cancer cells (93–96). Similar findings were obtained for the CXCL12-CXCR4 axis, when over-expression of CXCL12 or constitutively active CXCR4 have led to reduced E-cadherin levels, accompanied with up-regulation of slug, vimentin and fibronectin or with switch toward elevated expression of cadherin 11 (84, 97, 98). Mechanistic analyses indicated that over-expression of CXCL12 in breast tumor cells has led to E-cadherin reduction through activation of the NF-κB pathway (84) and by up-regulation of β-catenin expression (98). As before, CXCL12-CXCR4-induced EMT-related properties in the cancer cells were often accompanied by increased tumor cell migration or invasion (84, 97, 98).

In parallel to the EMT-inducing properties of chemokines, they also were implicated in up-regulation of other processes that can promote metastasis, such as the release of MMPs that facilitate cancer cell spreading through extracellular matrix (ECM) components during extravasation or intravasation in the course of tumor cell dissemination. For example, twist up-regulated the expression of functional MMPs by non-transformed and transformed breast cells through CXCL8 and CCL5 activities (99–102). Other chemokines (CXCL1, CCL9) were also connected to induction of MMPs in breast tumor cells (102, 103). Elevated production of functional MMP2 and MMP9 was detected in breast tumor cells following CXCL12 stimulation, in the context of CXCR4 expression (104, 105). Addressing the roles of CXCR7/ACKR3, the other CXCL12 receptor, the study of murine breast tumor cells demonstrated that CXCL12 has induced the functional expression of MMP9 through CXCR7/ACKR3 *in vitro* and that CXCR7/ACKR3 inhibition has led to reduced tumor growth and MMP9 expression in tumors *in vivo* (106). In contrast, the research of rat mammary adenocarcinoma cells demonstrated that the ability of CXCR4-over-expressing cells to degrade matrix was antagonized by simultaneous co-expression of CXCR7/ACKR3 (107).

### Chemoresistance and Endocrine Resistance

A major obstacle in cancer therapy is intrinsic resistance to therapy or resistance that is acquired due to many different mechanisms, some of which taking place in the cancer cells following their interactions with TME elements. Being a part of the TME, chemokine axes were found to increase chemoresistance and resistance to endocrine therapy. In line with the fact that CSCs often stand in the basis of resistance to therapy (75, 108, 109), chemokine activities that increase the CSC sub-population may eventually also reduce tumor cell response to treatments, and the two processes may thus be connected [as reported for example in (81)].

To date, key roles were identified in breast cancer for CXCR1/CXCR2 and their CXCL1/CXCL8 ligands in promoting resistance to chemotherapeutic drugs such as doxorubicin and paclitaxel. By taking different measures to modify the expression of chemokine receptors or of the chemokines themselves, evidence was provided to the ability of this chemokine axis to directly promote chemoresistance *in vitro* and in animal studies (34, 66, 110, 111). Actually, *in vivo* studies demonstrated the benefit of co-administration or sequential treatment by chemotherapy and by inhibitory measures directed to CXCR1/CXCR2 on the volume of breast tumors, on their ability to metastasize, on neovascularization and on repopulation of the tumors by drug-resistant cells (34, 66, 81, 110–112). Along these lines, a study by Massagués and colleagues demonstrated that CXCR2 inhibitors that were administered to mice prior and in the course of chemotherapy, sensitized the tumor cells to the cytotoxic effects of the drugs. This study has revealed a regulatory loop in which genotoxic stress created by chemotherapeutic drugs limited the survival of breast tumor cells, but the expression of tumor necrosis factor α (TNFα) was also increased and has led to elevated production of CXCL1/2 by the tumor cells; these chemokines recruited CXCR2-expressing CD11b+ Gr1+ myeloid cells which in turn acted *via* S100A8/9 factors to promote the viability of tumor cells that expressed CXCR2. Myeloid cells recruited by CXCL1/2 thereby enhanced viability and chemoresistance in the cancer cells (113). Other members of the chemokine receptor family, such as CCR5 and CXCR4, were also noted as chemoresistance-mediating factors in breast cancer, acting to increase DNA repair [CCR5; (83)] or to elevate tumor cell proliferation and reduce sensitivity to chemotherapeutic drugs through induction of interleukin 1 (IL-1) by MSCs [CXCR4; (114)].

In addition, chemokines were reported as potential regulators of endocrine therapy in breast cancer. It was recently demonstrated that CCL2 derived from TAMs has led to elevated endocrine resistance in luminal-A breast cancer cells, through the activation of the PI3K/Akt/mTOR cascade (115). Important roles for the CXCL12-CXCR4 axis in this aspect were also reported, demonstrating that CXCL12 has induced the activation of the two estrogen receptors—ERα and ERβ–and these processes were down-regulated when CXCR4 was inhibited (54). Moreover, this same study demonstrated that CXCR4 activation has led to increased ERβ activities in the presence of tamoxifen treatment, altogether suggesting that CXCL12-induced CXCR4 activation enabled ERβ to promote down-stream signaling that may overcome inhibition by endocrine therapy. Roles for CXCR4 in resistance to endocrine treatments were also demonstrated when CXCL12 administration has increased the volumes of tumors generated by luminal-A breast tumor cells in mice treated by the estrogen receptor antagonist Fulvestrant (116). Along these same lines, it was found that CXCR7/ACKR3 increased the stability of ERα and conferred insensitivity to tamoxifen in luminal-A breast cancer cells (117).

To conclude this part of the review, the findings presented above emphasize the significant involvement of chemokines in up-regulating multiple tumor-enhancing aspects, where they act directly on the cancer cells to promote many levels of the malignancy process. By promoting tumor cell proliferation and survival, CSC enrichment, EMT induction, MMP production and therapy resistance, chemokines can elevate cancer establishment at the primary site as well as tumor cell dissemination to remote organs and the generation of metastases.

## Atypical Chemokine Activities Exerted at the Tumor Microenvironment

### Immune Checkpoints and Their Blockade

As noted above, by virtue of their chemotactic properties toward leukocytes, chemokines have a strong impact on the content of immune and inflammatory cells at the TME, as has been broadly investigated and reviewed [e.g., (4–12)]. However, a relatively novel topic of research that is still in its early phases indicates that chemokines can impact immune activities not only by directly dictating the leukocyte landscape at tumor/metastatic sites but also by affecting aspects related to inhibitory immune checkpoints—such as the PD-1/PD-L1 axis—and their blockade.

In this respect, an interesting research aspect is the ability of chemokines to up-regulate or stabilize the expression of PD-L1 by tumor cells, thus indirectly reducing the efficacy of anti-tumor immune functions. For example, CXCL8, whose source was in gastric cancer-derived MSCs, has induced the expression of PD-L1 in gastric cancer cells. The process was mediated by STAT3 and mTOR activation, leading to tumor cell resistance against CD8+ T cell-mediated killing (118). Along these lines, CXCL5 that was secreted by CAFs promoted the expression of PD-L1 by several colorectal cancer cell lines; here, CXCL5 signals were transferred through CXCR2, leading to PD-L1 up-regulation *via* a PI3K-dependent process. In mouse models the potential relevance of these findings to tumor progression was supported by the fact that the expression of CXCR2 and p-Akt was coordinated with PD-L1 expression in the tumors, and by immune-suppressive activities of the CAFs (119). Evidence in the same direction was obtained in colorectal cancer, where macrophage-derived CCL5 acted through p65-STAT3 complexes that bound the COP9 signalosome promoter, giving rise to PD-L1 stabilization and up-regulation in the cancer cells. These CCL5-mediated activities have led to enhanced escape from T cell-mediated immune activities (120).

Similarly, chemokines can up-regulate the expression of inhibitory immune checkpoints by myeloid cells at the TME. For example, in gastric cancer CSF-2 elevated the production by macrophages of CXCL8, which then elevated PD-1 expression by TAMs, giving rise to inhibition of CD8+ T cell activities (121). Also, in a recent study it was demonstrated that CXCR2+ MDSCs that were recruited to mouse mammary tumors by ELR+ CXC chemokines such as CXCL1/2, up-regulated the expression of immune checkpoint molecules (e.g., PD-1, CTLA-4, LAG3) by CD4+ and CD8+ T cells; they have also induced T cell exhaustion, partly through interferon γ (IFNγ) (122).

The above findings demonstrate that chemokine activities can lead to elevated expression of molecules that participate in down-regulation of immune activities in cancer. This way, chemokines can reduce the efficacy of therapeutic approaches using ICBs in cancer; accordingly, it was suggested that inhibition of chemokine axes may potentiate the efficacy of ICBs and augment anti-tumor immune activities that restrain tumor growth and metastasis. Obviously, such chemokine/chemokine receptor-targeting modalities can affect not only immune checkpoint regulation by chemokines, but also the impact of chemokines on the leukocyte landscape at tumor/metastatic sites. Indeed, in gastric cancer tissue samples obtained following treatment by the CXCR1/2 inhibitor reparixin, reduced levels of proliferating tumor cells were noted, alongside with reduced presence of PD-L1+ macrophages and increased fraction of CD8+ T cells (121). In rhabdomyosarcoma, where MDSCs of the CXCR2+ CD11b+ Ly6G^high^ phenotype mediated local immune suppression, the efficacy of antibodies directed to PD-1 was augmented when tumor-bearing mice had myeloid cells deficient in CXCR2 (123). Essentially similar findings were noted in a mouse model of lung cancer, where PMN-MDSCs reduced T cell proliferation, and treatment of mice with antibodies to CXCL5—which is a key chemoattractant of such MDSCs—has reduced the proportion of PMN-MDSCs and elevated the efficacy of anti-PD-L1 in increasing the survival of mice (124).

Similar findings demonstrating the importance of chemokine-induced MDSC infiltration in regulating the efficacy of ICB activities were provided in a recent study of anti-PD-1-resistant gliomas. Here, the survival of mice was increased when the CCR2 antagonist CCX872 was used, and further improvement was obtained upon treatment with anti-PD-1 (125). Increased benefit in terms of tumor inhibition was also obtained by measures that down-regulated CCR1 or CCL5 activities, combined with ICBs directed to PD-1 or PD-L1; here again, major roles were revealed for TAMs and MDSCs as targets whose inhibition potentiates the activities of ICBs (126, 127).

In parallel, improved activities of ICBs upon chemokine/chemokine receptor inhibition, manifested by reduced presence of immuno-suppressive/myeloid cells and increased immune surveillance was noted when the CXCL12-CXCR4 axis was down-regulated. In a model of metastatic breast cancer, in which ICBs were combined with the CXCR4 inhibitor plerixafor (AMD3100), the drug had multiple effects including reduction of fibrosis and of Tregs alongside with increased infiltration of CTLs; also, the inhibition of CXCR4 by plerixafor increased the effect of dual treatment of mice by anti-PD-1 + anti-CTLA-4, in terms of metastatic inhibition and prolonged survival (128). Following their studies demonstrating that plerixafor decreased the intra-tumor infiltration of Tregs, Poznansky and colleagues have recently combined plerixafor with anti-PD-1 in ovarian cancer models. The joint inhibitory modality had higher efficacy than each measure alone in enhancing infiltration and function of effector T cells, increasing memory T cells, and reducing the presence of MDSCs in the tumors. Compared with treatment by each element alone, the combined therapy was more potent in inhibiting tumor growth and increasing survival of mice (129). Along the same lines, anti-PD-L1 synergized with the CXCR4-inhibiting drug plerixafor in killing tumor cells in a mouse pancreatic model (130). Additional reports have also provided evidence to the benefit provided by co-inhibition of the CXCL12-CXCR4 axis and ICBs in other animal model systems, through regulation of immune activities (131–133).

In this context, it is important to mention that chemokines can induce intra-tumor infiltration not only of deleterious leukocyte sub-population but also of immune cells that can exert anti-tumor activities. Under these circumstances, it is expected that the chemokines themselves, rather than their inhibition, will collaborate with ICBs and increase their potency. One such example was demonstrated by the cooperativity between CCL5—known as chemattractant of T effector cells (2)—and CXCL9 that can act through CXCR3 to recruit Th1 cells, CD8+ T cells and NK cells (2). In this study, it was found that tumor-derived CCL5 has recruited effector T cells to tumors; the release of IFNγ by T cells has increased the production of CXCL9 by macrophages, leading to increased immune surveillance of the tumors. Moreover, tumors that expressed CCL5 and CXCL9 were responsive to anti-PD-1 treatment, in contrast to tumors that did not (134). These findings illustrate the importance of non-ELR CXC chemokines such as CXCL9 and CXCL10 that act through CXCR3 to recruit anti-tumor immune cells and can also have anti-angiogenic activities. Although when CXCR3 is expressed by tumor cells its ligands may promote tumor growth (16, 135–137), many studies demonstrated that these chemokines exert immuno-angiostatic activities on the TME (16, 138, 139). Thus, it is expected that in different tumor systems, chemokines acting through CXCR3 would act alongside with ICB activities, as was suggested by several published reviews (16, 138, 139).

### Bone Remodeling

The bone is a preferred metastatic site which generally marks poor prognosis in many malignancies, including breast cancer. Following tumor cell invasion to bones, their metastatic colonization at the site is accompanied by bone remodeling, reflecting an inappropriate balance between bone-forming osteoblasts and bone-resorbing osteoclasts that leads to bone destruction. This osteolytic process, driven by several mediators such as RANKL and others, serves well the needs of the metastasizing cancer cells and contributes to their outgrowth in this niche (140, 141).

Many members of the chemokine family were found to contribute to bone remodeling with and without connection to malignancy (140–144). In this context, under physiological conditions, CXCL8 can promote RANKL production by osteoblasts and collaborate with it to increase the generation of osteoclasts (141, 145–147). Thus, when cancer cells acquire the ability to express CXCL8, it is assumed that they will enhance osteoclastogenesis during the metastatic process. Indeed, several studies support such a scenario: when breast tumor cell-derived supernatants promoted osteoclastogenesis, as indicated by increased generation of TRAP+ cells out of peripheral blood mononuclear cells, the process was down-regulated by inhibitors of CXCL8 or its receptors (146, 148, 149). Moreover, CXCL8 produced by tumor cells or by CXCL8-transgenic mice gave rise to elevated osteolysis *in vivo*, whereas antibodies to CXCL8 prevented bone damage and elevated the survival of mice (146). It was also found that breast tumor cells produced semaphorin D, which has increased CXCL8 production by osteoblasts and the levels of TRAP+ expressing cells *in vitro*. In parallel, *in vivo* studies indicated that shRNA-mediated inhibition of semaphorin D expression in breast tumor cells has led to reduced levels of metastasis and longer survival, accompanied by reduced formation of osteolytic skeletal lesions (147). In this context, it is interesting to note that analysis of plasma from breast cancer patients identified significant correlation between increased CXCL8 levels and elevated degree of bone resorption as well as with bone metastasis, supporting key roles for CXCL8 in this setting (146).

In parallel, CCL2 was found to be expressed at the site of metastatic breast cancer localization in the bones (150) and breast cancer-derived CCL2 has acted through CCR2 to promote osteoclast differentiation and contributed to bone metastasis (151). Moreover, it was found that MAPK11 (p38β) activation in breast cancer cells has given rise to elevated CCL2 production, which then contributed to increased bone resorption (152).

The picture seems to be more complex in the case of axes including CCL5 and CCL3, and their shared receptors CCR1 and CCR5 in regulating bone remodeling in cancer (141, 144, 153). Information also is lacking regarding the roles of the CXCL12-CXCR4 pair in this context. This axis is of particular interest because CXCL12 was found to promote bone resorption under physiological conditions, and in parallel is a leading factor in driving tumor cell homing to the bones in a very large number of malignancies. In view of these dual roles of CXCL12, it is expected that the CXCL12-CXCR4 pair will be instrumental in regulating osteoclastogenesis and osteolysis in tumors, but currently this aspect was mainly investigated in multiple myeloma (141, 144) and needs to be extensively addressed in future studies.

### Pro-cancerous Tumor-Stroma Interactions

MSCs and CAFs are major components of the tumor stroma that in many malignancy-related systems (including breast cancer), although not all, have been strongly connected to increased tumor-promoting functions. The activities of MSCs and CAFs at the tumor setting include induction of EMT, angiogenesis and more, and are affected by their interactions with the TME, primarily the pro-inflammatory TME (154–165).

The sources of CAFs are diverse, including resident fibroblasts, adipose MSCs and bone marrow-derived MSCs that differentiate to CAFs at the tumor site (155, 166–169). In addition to their roles as chemoattractants of MSCs to tumor sites, which have been reviewed previously [e.g., (155, 158, 170, 171)], chemokines can stand in the basis of tumor-stroma interactions that promote cancer progression. MSCs and CAFs can establish direct contacts with the cancer cells; in addition, the tumor cells and stromal cells can affect each other indirectly by the release of soluble mediators. In the scope of this article we hereby elaborate on studies demonstrating the roles of chemokines in regulating tumor-stroma interactions, which eventually affect the pro-malignancy functions of one or both cell types, or of the TME.

In this respect, our recent study indicated that interactions formed between TNBC cells and MSCs under the influence of the pro-inflammatory cytokine TNFα have given rise to increased lung metastasis in a breast cancer animal model system (159). In this system, we demonstrated that cell-to-cell contacts between the tumor cells and the stromal cells, as well as soluble mediators, have led to increased production of inflammatory chemokines; this process was further promoted by stimulation of tumor-stroma co-cultures with TNFα and IL-1β. One of the key chemokines that was potently elevated due to such inflammation-driven TNBC-MSC cross-talks was CXCL8. Our findings indicated that following TNFα stimulation of tumor-stroma co-cultures, NF-κB activation has led to CXCL8 induction, partly through a Notch1-dependent process. Then, CXCL8 that was expressed at elevated levels played direct roles in promoting angiogenesis as well as tumor cell migration and invasion (159, 160). Here, it was interesting to note that similar elevations in CXCL8 production were not evident when the partners in the co-cultures were luminal-A breast tumor cells instead of TNBC cells (159). In another study of TNBC cells, Jin et al. demonstrated that in response to factors released by the tumor cells, CAFs and macrophages released CXCL8 that promoted the proliferation and migration of the cancer cells in a process that depended on CXCR2 activation (40). Other members of the ELR+ CXC chemokine family, CXCL1 and CXCL2 were found to be induced in normal mammary fibroblasts that gained a CAF phenotype in response to tumor cell-derived osteopontin, that also promoted tumor growth (172).

In breast cancer it was also demonstrated that CCL2 levels were higher in stromal cells derived from tumors compared to normal breast tissues and that fibroblast-derived CCL2 contributed to tumor growth and metastasis *in vivo* (82, 162). More so, physical as well as indirect interactions between breast tumor cells and cancer-associated stromal cells (and not normal mammary stromal cells) have contributed to elevated levels of CCL2 (82, 159, 161, 162). It was found that under such interactive settings, CCL2 has contributed to elevated tumor cell migration, generation of CSCs and angiogenesis (82, 161). CCL2 production in the tumor-stroma setting was connected to pro-inflammatory conditions: pro-inflammatory stimuli (TNFα and IL-1β) have strongly up-regulated the release of CCL2 by tumor-stroma co-cultures (159), and in parallel CCL2 has induced an inflammatory TME in mice, demonstrated by high localization of macrophages and increased stroma and collagen density in mice (173). As with CXCL8, a connection to the Notch pathway was revealed for CCL2 in mediating tumor-stroma interactions, when CCL2 produced by fibroblasts that were activated in the presence of breast cancer cells has elevated CSC levels in cancer cells by activating the Notch pathway, and has induced the expression of Notch1 by the cancer cells (82).

Strong interactions through the CCL5-CCR5 axis were also reported to exist between breast tumor cells and stromal cells, mainly MSCs. For example, osteopontin was found to be a key factor released by breast tumor cells, binding αVβ3 integrins expressed by MSCs and then giving rise to elevated levels of CCL5 production. This interactive loop gave rise to increased metastasis in mice that were administered with tumor-MSC co-cultures, through osteopontin and CCL5-dependent mechanisms (163). Also, the study by Weinberg and colleagues demonstrated that CCL5 released by MSCs has acted through CCR5 to promote breast tumor cell migration, invasion and metastasis in animal studies (164). In the same spirit, CCL5 produced by MSCs has acted on CCR5-expressing breast tumor cells, leading to the release of CSF-1 and then to increased accumulation of macrophages and MDSCs in tumors. Accordingly, CCR5 inhibition by siRNAs gave rise to reduced metastasis formation, accompanied by decreased levels of CSF-1-expressing macrophages and CD11+ Ly6C+ MDSCs (174). In another study, it was demonstrated that the conditioned medium (CM) of MSCs increased the expression levels of two CCL5 receptors, CCR5 and CCR1 by murine breast tumor cells; in line with these findings the inhibitor met-CCL5 inhibited the migration of the cancer cells in response to MSC-derived CM (102). Cooperativity of CCL5 with IL-6 was also noted when CM of MSCs promoted breast tumor cell migration (175).

When coming to address the roles of CXCL12 in mediating tumor-stroma interactions in breast cancer, the majority of studies indicated that this chemokine or CXCR4 stand in the center of tumor-promoting cross-talks between cancer cells and stromal cells. CAFs constituted a major source for CXCL12, and produced it in higher levels than normal fibroblasts or fibroblasts located in seemingly healthy tissues that were adjacent to patient tumors (176–178). Moreover, CXCL12 production was elevated when CAFs or MSCs interacted directly or indirectly with breast tumor cells (86, 179). Under such conditions, CXCL12- and CXCR4-mediated signaling elevated a large number of pro-cancerous characteristics and functions in breast cancer: tumor cell proliferation and invasion, generation of CSCs and angiogenesis (through attraction of endothelial progenitor cells), as well as tumor growth and metastasis *in vivo* (86, 176–180).

However, the roles of CXCR4 in mediating tumor-stroma networks that promoted breast malignancy were put to question in several other studies. In one of these works it was demonstrated that CM of MSCs elevated the proliferation of breast tumor cells not through CXCR4, but rather *via* CXCR7/ACKR3 (181). Another study indicated that CXCR7/ACKR3 expression by breast cancer cells was down-regulated by MSC-derived CXCL12 (possibly due to ligand-dependent receptor internalization), and under these conditions, metastasis was reduced. However, when TGFβ was introduced, CXCL12 production by the MSCs was reduced, CXCR7/ACKR3 expression levels remained intact and metastasis was elevated (182). Here, it is worth noting that unlike these findings, a positive feedback loop between TGFβ and CXCL12 was found in relation to CAFs, when TGFβ and CXCL12 up-regulated each other's expression in mammary CAFs, and both contributed to the gradual process of fibroblast transition to CAFs (177).

Overall, the research on the impact of chemokines at the TME has been largely expanded beyond their fundamental roles in regulating the migration of leukocytes, endothelial cells and stromal cells. Currently, it is becoming evident that chemokines affect the ability of immune cells to exert anti-tumor activities by regulating the expression of immune checkpoints and the activity of ICBs. Moreover, chemokines facilitate metastasis by remodeling bone structure and by mediating pro-tumorigenic interactions that take place between cancer cells and stromal cells. Evidently, all of these activities largely contribute to elevated tumor progression and may lead to reduced patient survival.

### Activities of Atypical Chemokine Receptors in Cancer

Between others, in the previous sections of the article we described non-conventional activities of CXCL12, taking place *via* its two receptors, CXCR4 and CXCR7/ACKR3. Whereas, CXCR4 was characterized as a typical tumor-supporting receptor, many lines of evidence indicated CXCR7/ACKR3 can have pro-metastatic effects but in specific settings it can act in an opposite manner. The tumor-restricting activities of CXCR7/ACKR3 may be connected to the fact that unlike CXCR4, it is an atypical chemokine receptor (and thus was given the additional name ACKR3). ACKRs lack the classical heterotrimeric G protein-mediated signaling pathway, they control responses to a variety of CXC and CC chemokines and they are expressed by various cell types. This class of receptors was originally considered as “decoy receptors” that sequester chemokines from the microenvironment, thereby inhibiting the effects of chemokines at different settings. In parallel, recent studies indicate that ACKRs regulate cancer progression by their chemokine-sequestering functions, as well as by other mechanisms.

Aside from controlling motility-related aspects in cancer, such as tumor cell invasion, endothelial cell migration (angiogenesis) and eventually tumor progression *in vivo* (183–187), CXCR7/ACKR3 regulates non-conventional cancer-related activities. The array of atypical cancer-regulating functions of CXCR7/ACKR3—when it acted alone or in the context of CXCR4—were discussed in the previous sections of the article, as appropriate. The intriguing findings on CXCR7/ACKR3 illustrate the importance of the ACKR subgroup in general; thus in this section of the manuscript we discuss the atypical roles of additional key ACKRs in malignancy: ACKR1 (DARC, Duffy), ACKR2 (D6, CCBP2) and ACKR4 (CCRL1, CCX-CKR).

ACKR1 is a highly promiscuous receptor that binds a large number of chemokines, from the CC and CXC sub-families, mainly those of the inflammatory sub-group. Through internalization, ACKR1 plays key roles as depot of chemokines; accordingly, its constitutive expression by venular endothelial cells results in low availability of ELR+ CXC chemokines that promote angiogenesis (2, 4, 27). By sequestering ELR+ CXC chemokines as well other members of the family, and possibly also *via* other pathways, ACKR1 usually acquires anti-tumorigenic effects. Indeed, ACKR1 was strongly connected to improved outcomes in breast cancer as well as in several other malignancies, at times upon co-expression with ACKR2 or ACKR4 (188–192). Accordingly, ACKR1 was causatively linked to reduced tumor growth and metastasis in animal models, and ACKR1 single nucleotide polymorphisms that were related to chemokine sequestration affected angiogenesis, tumorigenesis and lung metastasis (193–195). The anti-tumor activities of ACKR1 were mediated not only by reducing microvessel density, but also by inhibiting atypical cancer-related activities, such as MMP9 production and tumor cell proliferation, as well as increasing tumor cell senescence (192, 194–196); from the mechanistic perspective, it was demonstrated that ACKR1 caused anti-tumor effects by interfering with CXCR2-induced STAT3 activation in pancreatic adenocarcinoma cells (192). Moreover, in prostate cancer ACKR1 expressed by vascular endothelial cells interacted with tetraspanin KAI1 (CD82) on tumor cells, leading to decreased DNA synthesis and induction of tumor cell senescence (195); in parallel, it was found that melanoma-expressed KAI interacted with endothelial cell-expressed ACKR1 preventing CXCL8-inudced gap formation in endothelial cells and leading to tumor cell senescence (197).

Very much like ACKR1, ACKR2 binds and internalizes inflammatory chemokines, leading them to degradation; however, unlike ACKR1, the activities of ACKR2 are limited mostly to CC chemokines that signal through CCR1 and CCR5 (2, 4, 27). By virtue of its expression by lymphatic endothelial cells and by tumor cells, ACKR2 plays key roles in preventing inflammatory conditions in a variety of settings, and was mainly referred to as tumor-restricting receptor (2, 4, 27). In breast cancer and in many other malignancies, ACKR2 expression was causatively linked to down-regulation of tumor growth and metastasis (198–202). In many cases, ACKR2 inhibited the infiltration of tumor-supporting leukocytes, angiogenesis or tumor cell invasion; often, these processes were accompanied by reduced levels of the relevant chemokines and competition with CCR2-mediated signaling (198–202). However, through the same mechanisms of CCL2/CCR2 inhibition, ACKR2 was also reported to prevent the activities of beneficial leukocyte sub-populations, such as NK cells and neutrophils that are cytotoxic against tumor cells (203, 204).

By functioning in these manners, the tumor-restricting but also the tumor-promoting activities of ACKR2 resulted from the expected, motility-related functions that are involved in cancer progression. However, in addition, ACKR2 was found to restrict tumor progression by regulating atypical chemokine activities at the tumor setting, such as cancer cell proliferation (198, 199). However, in this context of non-conventional cancer-related chemokine activities, it is possible that ACKR2 may also have pro-tumor effects. This is illustrated by a recent study demonstrating that fibroblast-derived CXCL14 acted in the context of tumor cell expressed ACKR2, activating the ERK pathway and inducing EMT, elevating migration and lung colonization by luminal-A breast cancer cells (205).

ACKR4 joins ACKR2 in sequestering CC chemokines, but with preference to homeostatic members of the family: CCL19, CCL21, CCL25 (and with lower affinity CCL13). Resembling ACKR1 and many of the functions of ACKR2, ACKR4 demonstrates predominantly tumor-restricting effects, and was positively correlated with patient survival rates in several cancers; at times, ACKR4 expression was inversely correlated with the expression of chemokines in patient materials (188, 191, 206–208). Supporting these findings are the results of a breast cancer report, demonstrating that ACKR4 overexpression by breast tumor cells inhibited tumor growth and lung metastases, and decreased the expression of mouse CCL19, CCL21, CCL25 and CXCL13 chemokines in xenografts (208). In this study, and in reports on hepatocellular carcinoma and nasopharyngeal carcinoma, the tumor-restricting effects of ACKR4 were mediated by inhibition of cancer cell proliferation, EMT and/or migration, through abrogation of the relevant chemokine axes (207–209). Conversely, although smaller primary tumors were formed when CXCR4-over-expressing mouse TNBC cells were administered to mice, the cancer cells acquired increased ability to colonize the lungs; in this case, ACKR4 promoted EMT in the tumor cells, reduced adherence of the cancer cells to each other and to ECM proteins, and increased their resistance to anoikis (210).

The findings discussed above suggest that CXCR7/ACKR3 may have definite tumor-enhancing roles but also can acquire anti-malignancy effects in certain settings. In contrast, ACKR1, ACKR4 and in many cases also ACKR2 exert anti-tumor functions at conventional migration-related processes as well as at non-conventional aspects. Thus, specific ACKRs may have important implications toward chemokine-designed therapies in cancer.

## Discussion

The chemokine family includes a very large number of members, which regulate physiological and pathological conditions at many different levels. In cancer, specific members of the family that act under defined situations can exert anti-tumor activities, e.g., by recruiting cytotoxic immune cells to tumors or down-regulating angiogenesis. However, in many of the malignancies, a large number of the chemokines demonstrates the ability to promote tumor growth and progression and dominate the setting by giving rise to elevated tumor aggressiveness.

**Figure 1:**
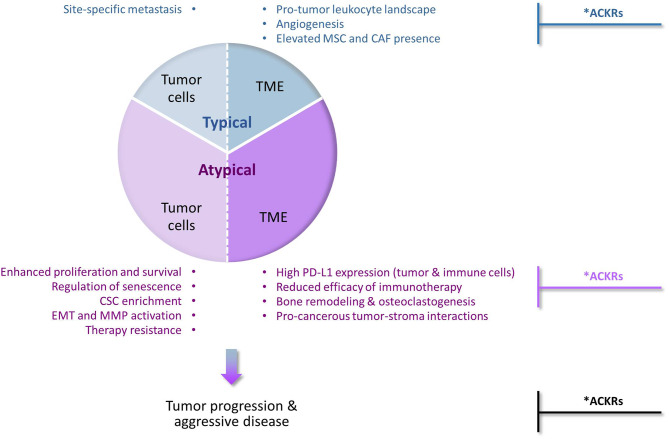
Typical and atypical pro-tumor activities of chemokines and their receptors in cancer. The chemokine family contains many different members, some of which can limit tumor progression for example by inducing the recruitment of cytotoxic immune cells to tumors, or by inducing angiostasis (e.g., CXCR3). However, extensive investigations of chemokine roles in cancer indicate that chemokine activities that promote tumor development and progression are very common and often dominate the malignancy process. Being prime regulators of leukocyte migration in the immune context, chemokines are primarily considered as inducers of cellular motility. Accordingly, chemokine activities that promote tumor progression *via* induction of directional cell motility—of leukocytes, endothelial cells, stromal cells and cancer cells—are regarded in the scope of this review as “Typical”. Very much like the “Typical” chemokine activities, also those that do not directly affect cellular motility and are thus termed herein “Atypical”, can be exerted on the tumor cells or on the TME (tumor microenvironment). By addressing most of these aspects in breast cancer, we emphasize in this review article the atypical activities of chemokines in cancer (thus given a higher proportion in this drawing, but not necessarily so in the actual cancer setting). In the “Typical” part, we mention that typical chemokine-induced migration can lead to homing of cancer cells at specific metastatic sites and to remodeling of the tumor landscape by recruiting leukocytes, inducing angiogenesis through endothelial cell migration, and attracting MSCs that can then differentiate to CAFs. In parallel, in the “Atypical” part, we describe the roles of chemokines in reinforcing (1) the aggressiveness of the tumor cells, by elevating tumor cell proliferation and survival, regulating senescence, enriching tumors for CSCs, inducing EMT and MMP production and elevating resistance to chemotherapy and endocrine treatments; and (2) the pro-metastatic nature of the TME, by interfering with the activities of ICBs, remodeling the bone niche by elevating osteoclastogenesis and bone resorption, and promoting tumor-stroma interactions that contribute to elevated malignancy. Together, all of these chemokine effects—typical and atypical—eventually lead to elevated metastasis and worsening of disease course. *Anti-metastatic activities in cancer: Under specific settings, the pro-metastatic activities of chemokines can be inhibited by other chemokines that act through classical chemokine receptors (e.g., CXCR3) or by atypical chemokine receptors (ACKRs). Such tumor-inhibitory activities of ACKRs have been well-documented for ACKR1 and ACKR4, whereas ACKR2 was mostly reported as an anti-malignancy element, with pro-tumor activities reported as well. In contrast, CXCR7/ACKR3 is mainly characterized as tumor-enhancing factor, although its roles in malignancy are complex, can be anti-tumorigenic and often reflect its interactions with CXCR4, the other receptor that binds CXCL12.

Their prime function as inducers of cellular motility has set chemokines and their receptors as major regulators of malignancy-related events that depend on cell migration in response to chemotactic signals (Figure 1—“Typical” chemokine activities in cancer). These events include primarily the following mechanisms: (1) Chemokines control in spatial and temporal manners the migration of different leukocyte subsets and their recruitment to tumors/metastases, thus having a strong impact on leukocyte content at these sites. Accordingly, the equilibrium between immune cells that recognize tumor antigens *vs*. pro-inflammatory/immune-suppressive cells has major roles in determining the fate of the developing tumor and of metastases; (2) Signals delivered by specific chemokines promote the migration of endothelial cells and their progenitors, thus supporting the essential process of angiogenesis; (3) Chemokines recruit MSCs from other tissues, primarily bones, to tumors/metastases; there, the MSCs can express many tumor-promoting activities on their own, and also after their transition to CAFs; (4) Cancer cells that express chemokine receptors respond to their corresponding chemotactic cues at remote sites, thus chemokines form an important venue that through directed tumor cell migration dictates site-specific metastasis.

At first, reports on chemokine activities that are beyond regulation of cell motility were sporadic; however, with time it became clear that chemokines influence cancer cells and the TME at many levels that are not directly connected to cell migration, eventually supporting the establishment of primary tumors and cancer cell spreading to metastatic sites. Such chemokine functions were exemplified in this article by focusing on the activities of several tumor-promoting chemokines of the CXC and CC sub-families—mainly of the inflammatory arm—in breast cancer (Figure 1—“Atypical” chemokine activities in cancer). We have illustrated such atypical roles of chemokines in promoting tumor cell proliferation and survival and in parallel in regulating the senescence of cancer cells; in enriching tumors with CSCs; in promoting the mesenchymal phenotype of cancer cells (EMT) and the release of MMPs; and in elevating resistance to chemotherapy and to endocrine therapy. In parallel, atypical chemokine activities lay out the basis for a tumor-supportive TME by modulating immune checkpoints and interfering with their blockade, by facilitating bone metastasis through osteoclastogenesis and bone resorption, and by mediating tumor-stroma interactions that promote the pro-cancerous potential of the tumor cells and of their adjacent milieu.

Very likely, the different levels affected by the chemokines are inter-connected, further amplifying disease progression. First and foremost is the strong connection of chemokines to immune activities: here, the ability of chemokines to regulate the immune and inflammatory contextures of tumors is joined by their ability to promote the expression of inhibitory immune checkpoints and to regulate the efficacy of their blockade. As a result of these joint activities, chemokines may strongly impact the efficacy of ICBs and of other immune-mediated anti-cancer therapies.

Another illustration of integrative chemokine effects at several levels simultaneously is provided when chemokines affect in atypical manners the cancer cells themselves. For example, tumor cell stemness is strongly connected to elevated EMT and to therapy resistance; accordingly, in some of the studies mentioned above chemokines were found to promote some of these processes concurrently. Similarly, when cancer cells acquire in response to chemokine activities a mesenchymal phenotype that is manifested by EMT-related properties, they often also gain increased ability to migrate and invade. Additional strong connections are revealed when chemokines stand in the center of tumor-stroma interactions. Such interactions, which are mediated by chemokines or lead to their increased production can eventually play key roles in promoting directly the aggressiveness of the cancer cells (proliferation, invasion, therapy resistance *etc*.) and the pro-tumor nature of the TME (for example, recruitment of inflammatory cells, angiogenesis and bone remodeling).

The research of some of these topics is only at its beginning, and evidence of novel aspects that are regulated by chemokines in the course of cancer development and progression are now emerging. These aspects include for example the ability of chemokines to elevate the levels of DNA repair (83), to alter tumor cell metabolism (48, 96, 211, 212), to regulate the localization and retention of dormant cancer cells in the bone marrow (213) and to promote vasculogenic mimicry by tumor cells (38).

The tumor-promoting roles of chemokines in malignancy—through conventional (motility-related) and non-conventional functions—should be carefully considered in the context of tumor heterogeneity. Malignant diseases differ considerably from each other in terms of cause and progression patterns; this is illustrated not only when different cancer types are compared but also within the same disease, as is the case in breast cancer (e.g., the TNBC *vs*. luminal-A subtypes). In addition, a very challenging aspect in this regard is intra-tumor heterogeneity which is observed in many tumor types (73). Obviously, when chemokine roles in cancer are investigated, these aspects of inter-tumor and intra-tumor heterogeneity need to be considered.

More so, chemokine roles in cancer and their relevance for therapy need to be regarded in the broader scope of “chemokine heterogeneity”. Here, one needs to consider tumors and metastases as multi-chemokine organs, thus the impact of chemokines on tumor progression depends much on their relative amounts, temporal/spatial localization at the tumor site/metastatic organs and the expression of corresponding receptors by cancer cells, leukocytes, endothelial cells, and stromal cells. Eventually, these parameters will dictate to a large degree which of the chemokine/s will dominate the overall malignant setting, *via* its/their typical and atypical activities, affecting the tumor cells or the TME.

The aspect of “chemokine receptor heterogeneity” adds even more to the complexity of chemokine roles in cancer, by demonstrating the ability of ACKRs to control cancer-related activities. Whereas, CXCR7/ACKR3 has predominantly pro-metastatic roles in cancer, ACKR1 and ACKR4 demonstrate mainly tumor-restricting effects. Here, they very often sequester and thus prevent the activities of pro-metastatic chemokines at many levels, conventional and non-conventional. Thus, certain ACKRs may represent a balancing arm of the chemokine field in controlling cancer progression and in this regard, should be considered as a therapeutic tool in cancer.

To conclude, our understanding of the roles of chemokines in cancer progression has been largely expanded with time. There are circumstances in which chemokines can interfere with the malignancy cascade, as illustrated by the immune-angiostasis functions of non-ELR CXC chemokines and by the tumor-restricting activities of ACKRs in certain settings. However, often the motility-driven and atypical activities of chemokines dominate the scene, leading to enhanced disease course and poor prognosis. Pre-clinical and initial clinical studies suggest that inhibitors of defined chemokines or of their receptors may be effective as therapeutic measures in cancer, primarily when they are joined by other modalities such as chemotherapy or ICBs [as illustrated above and also discussed in (4, 20, 21, 214–219)]; however, to reach the point in which chemokines or their receptors are used as targets in cancer therapy, extensive research of their functions, typical and atypical, is needed in the broader context of tumor heterogeneity, chemokine heterogeneity, and chemokine receptor heterogeneity at the tumor bed and in metastases.

## Author Contributions

DM and NE are equal contributors in bibliography search and in preparing defined article sections. AB-B was responsible for the entire setup and structure design of the manuscript, and contributed to all stages of manuscript preparation.

## Conflict of Interest

The authors declare that the research was conducted in the absence of any commercial or financial relationships that could be construed as a potential conflict of interest.

## References

[1] Griffith JW Sokol CL Luster AD. Chemokines and chemokine receptors: positioning cells for host defense and immunity. Annu Rev Immunol. (2014) 32:659–702. 10.1146/annurev-immunol-032713-120145 24655300

[2] Bachelerie F Ben-Baruch A Burkhardt AM Combadiere C Farber JM Graham GJ. International union of basic and clinical pharmacology. [corrected] LXXXIX Update on the extended family of chemokine receptors and introducing a new nomenclature for atypical chemokine receptors. Pharmacol Rev. (2014) 66:1–79. 10.1124/pr.113.007724 24218476 PMC3880466

[3] Chen K Bao Z Tang P Gong W Yoshimura T Wang JM. Chemokines in homeostasis and diseases. Cell Mol Immunol. (2018) 15:324–34. 10.1038/cmi.2017.134 29375126 PMC6052829

[4] Mollica Poeta V Massara M Capucetti A Bonecchi R. Chemokines and chemokine receptors: new targets for cancer immunotherapy. Front Immunol. (2019) 10:379. 10.3389/fimmu.2019.00379 30894861 PMC6414456

[5] Do HTT Lee CH Cho J. Chemokines and their receptors: multifaceted roles in cancer progression and potential value as cancer prognostic markers. Cancers. (2020) 12:287. 10.3390/cancers12020287 31991604 PMC7072521

[6] Shalapour S Karin M. Pas de deux: control of anti-tumor immunity by cancer-associated inflammation. Immunity. (2019) 51:15–26. 10.1016/j.immuni.2019.06.021 31315033 PMC6640850

[7] Del Prete A Schioppa T Tiberio L Stabile H Sozzani S. Leukocyte trafficking in tumor microenvironment. Curr Opin Pharmacol. (2017) 35:40–7. 10.1016/j.coph.2017.05.004 28577499

[8] Colotta F Allavena P Sica A Garlanda C Mantovani A. Cancer-related inflammation, the seventh hallmark of cancer: links to genetic instability. Carcinogenesis. (2009) 30:1073–81. 10.1093/carcin/bgp127 19468060

[9] Becht E Giraldo NA Germain C de Reynies A Laurent-Puig P Zucman-Rossi J. Immune contexture, immunoscore, and malignant cell molecular subgroups for prognostic and theranostic classifications of cancers. Adv Immunol. (2016) 130:95–190. 10.1016/bs.ai.2015.12.002 26923001

[10] Taube JM Galon J Sholl LM Rodig SJ Cottrell TR Giraldo NA. Implications of the tumor immune microenvironment for staging and therapeutics. Mod Pathol. (2018) 31:214–34. 10.1038/modpathol.2017.156 29192647 PMC6132263

[11] Shaul ME Fridlender ZG. Neutrophils as active regulators of the immune system in the tumor microenvironment. J Leukoc Biol. (2017) 102:343–9. 10.1189/jlb.5MR1216-508R 28264904

[12] Ben-Meir K Twaik N Baniyash M. Plasticity and biological diversity of myeloid derived suppressor cells. Curr Opin Immunol. (2018) 51:154–61. 10.1016/j.coi.2018.03.015 29614426

[13] Borsig L Wolf MJ Roblek M Lorentzen A Heikenwalder M. Inflammatory chemokines and metastasis-tracing the accessory. Oncogene. (2013) 33:3217–24. 10.1038/onc.2013.272 23851506

[14] Todorovic-Rakovic N Milovanovic J. Interleukin-8 in breast cancer progression. J Interferon Cytokine Res. (2013) 33:563–70. 10.1089/jir.2013.0023 23697558 PMC3793647

[15] Alfaro C Sanmamed MF Rodriguez-Ruiz ME Teijeira A Onate C Gonzalez A. Interleukin-8 in cancer pathogenesis, treatment and follow-up. Cancer Treat Rev. (2017) 60:24–31. 10.1016/j.ctrv.2017.08.004 28866366

[16] Susek KH Karvouni M Alici E Lundqvist A. The Role of CXC Chemokine receptors 1-4 on immune cells in the tumor microenvironment. Front Immunol. (2018) 9:2159. 10.3389/fimmu.2018.02159 30319622 PMC6167945

[17] Reynders N Abboud D Baragli A Noman MZ Rogister B Niclou SP. The distinct roles of CXCR3 variants and their ligands in the tumor microenvironment. Cells. (2019) 8:613. 10.3390/cells8060613 31216755 PMC6627231

[18] Keeley EC Mehrad B Strieter RM. Chemokines as mediators of tumor angiogenesis and neovascularization. Exp Cell Res. (2011) 317:685–90. 10.1016/j.yexcr.2010.10.020 21040721 PMC3073599

[19] Salazar N Zabel BA. Support of tumor endothelial cells by chemokine receptors. Front Immunol. (2019) 10:147. 10.3389/fimmu.2019.00147 30800123 PMC6375834

[20] Argyle D Kitamura T. Targeting macrophage-recruiting chemokines as a novel therapeutic strategy to prevent the progression of solid tumors. Front Immunol. (2018) 9:2629. 10.3389/fimmu.2018.02629 30483271 PMC6243037

[21] Jiao X Nawab O Patel T Kossenkov AV Halama N Jaeger D. Recent advances targeting CCR5 for cancer and its role in immuno-oncology. Cancer Res. (2019) 79:4801–7. 10.1158/0008-5472.CAN-19-1167 31292161 PMC6810651

[22] Lacalle RA Blanco R Carmona-Rodriguez L Martin-Leal A Mira E Manes S. Chemokine receptor signaling and the hallmarks of cancer. Int Rev Cell Mol Biol. (2017) 331:181–244. 10.1016/bs.ircmb.2016.09.011 28325212

[23] Ben-Baruch A. Site-specific metastasis formation: chemokines as regulators of tumor cell adhesion, motility and invasion. Cell Adh Migr. (2009) 3:328–33. 10.4161/cam.3.4.9211 19550136 PMC2802740

[24] Kitamura T Pollard JW Therapeutic potential of chemokine signal inhibition for metastatic breast cancer. Pharmacol Res. (2015) 100:266–70. 10.1016/j.phrs.2015.08.004 26275794 PMC4617477

[25] Zlotnik A Burkhardt AM Homey B. Homeostatic chemokine receptors and organ-specific metastasis. Nat Rev Immunol. (2011) 11:597–606. 10.1038/nri3049 21866172

[26] Jacquelot N Duong CPM Belz GT Zitvogel L. Targeting chemokines and chemokine receptors in melanoma and other cancers. Front Immunol. (2018) 9:2480. 10.3389/fimmu.2018.02480 30420855 PMC6215820

[27] Massara M Bonavita O Mantovani A Locati M Bonecchi R Atypical chemokine receptors in cancer: friends or foes? J Leukoc Biol. (2016) 99:927–33. 10.1189/jlb.3MR0915-431RR 26908826

[28] Eroles P Bosch A Perez-Fidalgo JA Lluch A. Molecular biology in breast cancer: intrinsic subtypes and signaling pathways. Cancer Treat Rev. (2012) 38:698–707. 10.1016/j.ctrv.2011.11.005 22178455

[29] Gerratana L Fanotto V Bonotto M Bolzonello S Andreetta C Moroso S. Pattern of metastatic spread and prognosis of breast cancer biologic subtypes. J Clin Oncol. (2014) 32:125–33. 10.1200/jco.2014.32.15_suppl.e1253225630269

[30] Payne AS Cornelius LA. The role of chemokines in melanoma tumor growth and metastasis. J Invest Dermatol. (2002) 118:915–22. 10.1046/j.1523-1747.2002.01725.x 12060384

[31] Hayashi S Kurdowska A Cohen AB Stevens MD Fujisawa N Miller EJ. A synthetic peptide inhibitor for alpha-chemokines inhibits the growth of melanoma cell lines. J Clin Invest. (1997) 99:2581–7. 10.1172/JCI119446 9169487 PMC508103

[32] Fujisawa N Hayashi S Miller EJ. A synthetic peptide inhibitor for alpha-chemokines inhibits the tumour growth and pulmonary metastasis of human melanoma cells in nude mice. Melanoma Res. (1999) 9:105–14. 10.1097/00008390-199904000-00001 10380932

[33] Schadendorf D Moller A Algermissen B Worm M Sticherling M Czarnetzki BM. IL-8 produced by human malignant melanoma cells *in vitro* is an essential autocrine growth factor. J Immunol. (1993) 151:2667–75. 8360485

[34] Shao N Chen LH Ye RY Lin Y Wang SM. The depletion of interleukin-8 causes cell cycle arrest and increases the efficacy of docetaxel in breast cancer cells. Biochem Biophys Res Commun. (2013) 431:535–41. 10.1016/j.bbrc.2013.01.022 23321310

[35] Brandolini L Cristiano L Fidoamore A De Pizzol M Di Giacomo E Florio TM. Targeting CXCR1 on breast cancer stem cells: signaling pathways and clinical application modelling. Oncotarget. (2015) 6:43375–94. 10.18632/oncotarget.6234 26517518 PMC4791238

[36] Romero-Moreno R Curtis KJ Coughlin TR Miranda-Vergara MC Dutta S Natarajan A. The CXCL5/CXCR2 axis is sufficient to promote breast cancer colonization during bone metastasis. Nat Commun. (2019) 10:4404. 10.1038/s41467-019-12108-6 31562303 PMC6765048

[37] Hartman ZC Poage GM den Hollander P Tsimelzon A Hill J Panupinthu N. Growth of triple-negative breast cancer cells relies upon coordinate autocrine expression of the proinflammatory cytokines IL-6 and IL-8. Cancer Res. (2013) 73:3470–80. 10.1158/0008-5472.CAN-12-4524-T 23633491 PMC3853111

[38] Aikins AR Kim M Raymundo B Kim CW. Downregulation of transgelin blocks interleukin-8 utilization and suppresses vasculogenic mimicry in breast cancer cells. Exp Biol Med. (2017) 242:573–83. 10.1177/1535370216685435 28058861 PMC5685257

[39] Khazali AS Clark AM Wells A. Inflammatory cytokine IL-8/CXCL8 promotes tumour escape from hepatocyte-induced dormancy. Br J Cancer. (2018) 118:566–76. 10.1038/bjc.2017.414 29169181 PMC5830588

[40] Jin K Pandey NB Popel AS. Crosstalk between stromal components and tumor cells of TNBC via secreted factors enhances tumor growth and metastasis. Oncotarget. (2017) 8:60210–22. 10.18632/oncotarget.19417 28947965 PMC5601133

[41] Riverso M Kortenkamp A Silva E. Non-tumorigenic epithelial cells secrete MCP-1 and other cytokines that promote cell division in breast cancer cells by activating ERalpha via PI3K/Akt/mTOR signaling. Int J Biochem Cell Biol. (2014) 53:281–94. 10.1016/j.biocel.2014.05.023 24878609

[42] Han R Gu S Zhang Y Luo A Jing X Zhao L. Estrogen promotes progression of hormone-dependent breast cancer through CCL2-CCR2 axis by upregulation of Twist via PI3K/AKT/NF-kappaB signaling. Sci Rep. (2018) 8:9575. 10.1038/s41598-018-27810-6 29934505 PMC6015029

[43] Fang WB Jokar I Zou A Lambert D Dendukuri P Cheng N. CCL2/CCR2 chemokine signaling coordinates survival and motility of breast cancer cells through Smad3 protein- and p42/44 mitogen-activated protein kinase (MAPK)-dependent mechanisms. J Biol Chem. (2012) 287:36593–608. 10.1074/jbc.M112.365999 22927430 PMC3476325

[44] Yao M Fang W Smart C Hu Q Huang S Alvarez N. CCR2 chemokine receptors enhance growth and cell-cycle progression of breast cancer cells through SRC and PKC activation. Mol Cancer Res. (2019) 17:604–17. 10.1158/1541-7786.MCR-18-0750 30446625 PMC6359961

[45] Brummer G Acevedo DS Hu Q Portsche M Fang WB Yao M. Chemokine signaling facilitates early-stage breast cancer survival and invasion through fibroblast-dependent mechanisms. Mol Cancer Res. (2018) 16:296–308. 10.1158/1541-7786.MCR-17-0308 29133591 PMC5805627

[46] Murooka TT Rahbar R Fish EN. CCL5 promotes proliferation of MCF-7 cells through mTOR-dependent mRNA translation. Biochem Biophys Res Commun. (2009) 387:381–6. 10.1016/j.bbrc.2009.07.035 19607806

[47] Zhang Y Yao F Yao X Yi C Tan C Wei L. Role of CCL5 in invasion, proliferation and proportion of CD44+/CD24- phenotype of MCF-7 cells and correlation of CCL5 and CCR5 expression with breast cancer progression. Oncol Rep. (2009) 21:1113–21. 10.3892/or_00000331 19288016

[48] Gao D Rahbar R Fish EN. CCL5 activation of CCR5 regulates cell metabolism to enhance proliferation of breast cancer cells. Open Biol. (2016) 6:160122. 10.1098/rsob.160122 27335323 PMC4929946

[49] Velasco-Velazquez M Jiao X De La Fuente M Pestell TG Ertel A Lisanti MP. CCR5 antagonist blocks metastasis of basal breast cancer cells. Cancer Res. (2012) 72:3839–50. 10.1158/0008-5472.CAN-11-3917 22637726

[50] Pervaiz A Zepp M Mahmood S Ali DM Berger MR Adwan H. CCR5 blockage by maraviroc: a potential therapeutic option for metastatic breast cancer. Cell Oncol. (2019) 42:93–106. 10.1007/s13402-018-0415-3 30456574 PMC12994360

[51] Jin K Pandey NB Popel AS. Simultaneous blockade of IL-6 and CCL5 signaling for synergistic inhibition of triple-negative breast cancer growth and metastasis. Breast Cancer Res. (2018) 20:54. 10.1186/s13058-018-0981-3 29898755 PMC6000947

[52] Kishimoto H Wang Z Bhat-Nakshatri P Chang D Clarke R Nakshatri H. The p160 family coactivators regulate breast cancer cell proliferation and invasion through autocrine/paracrine activity of SDF-1alpha/CXCL12. Carcinogenesis. (2005) 26:1706–15. 10.1093/carcin/bgi137 15917309

[53] Pattarozzi A Gatti M Barbieri F Wurth R Porcile C Lunardi G. 17beta-estradiol promotes breast cancer cell proliferation-inducing stromal cell-derived factor-1-mediated epidermal growth factor receptor transactivation: reversal by gefitinib pretreatment. Mol Pharmacol. (2008) 73:191–202. 10.1124/mol.107.039974 17959712

[54] Sauve K Lepage J Sanchez M Heveker N Tremblay A. Positive feedback activation of estrogen receptors by the CXCL12-CXCR4 pathway. Cancer Res. (2009) 69:5793–800. 10.1158/0008-5472.CAN-08-4924 19584281

[55] Salazar N Munoz D Kallifatidis G Singh RK Jorda M Lokeshwar BL. The chemokine receptor CXCR7 interacts with EGFR to promote breast cancer cell proliferation. Mol Cancer. (2014) 13:198. 10.1186/1476-4598-13-198 25168820 PMC4167278

[56] Tang X Li X Li Z Liu Y Yao L Song S. Downregulation of CXCR7 inhibits proliferative capacity and stem cell-like properties in breast cancer stem cells. Tumour Biol. (2016) 37:13425–33. 10.1007/s13277-016-5180-1 27460092

[57] Gao W Mei X Wang J Zhang X Yuan Y. ShRNA-mediated knock-down of CXCR7 increases TRAIL-sensitivity in MCF-7 breast cancer cells. Tumour Biol. (2015) 36:7243–50. 10.1007/s13277-015-3432-0 25894375

[58] Luker KE Lewin SA Mihalko LA Schmidt BT Winkler JS Coggins NL. Scavenging of CXCL12 by CXCR7 promotes tumor growth and metastasis of CXCR4-positive breast cancer cells. Oncogene. (2012) 31:4750–8. 10.1038/onc.2011.633 22266857 PMC3337948

[59] Yang M Zeng C Li P Qian L Ding B Huang L. Impact of CXCR4 and CXCR7 knockout by CRISPR/Cas9 on the function of triple-negative breast cancer cells. Onco Targets Ther. (2019) 12:3849–58. 10.2147/OTT.S195661 31190884 PMC6527053

[60] Boudot A Kerdivel G Habauzit D Eeckhoute J Le Dily F Flouriot G. Differential estrogen-regulation of CXCL12 chemokine receptors, CXCR4 and CXCR7, contributes to the growth effect of estrogens in breast cancer cells. PLoS ONE. (2011) 6:e20898. 10.1371/journal.pone.0020898 21695171 PMC3112227

[61] Hernandez-Segura A Nehme J Demaria M. Hallmarks of cellular senescence. Trends Cell Biol. (2018) 28:436–53. 10.1016/j.tcb.2018.02.001 29477613

[62] Kim YH Park TJ. Cellular senescence in cancer. BMB Rep. (2019) 52:42–6. 10.5483/BMBRep.2019.52.1.295 30526772 PMC6386235

[63] Acosta JC O'Loghlen A Banito A Guijarro MV Augert A Raguz S. Chemokine signaling via the CXCR2 receptor reinforces senescence. Cell. (2008) 133:1006–18. 10.1016/j.cell.2008.03.038 18555777

[64] Calcinotto A Kohli J Zagato E Pellegrini L Demaria M Alimonti A. Cellular senescence: aging, cancer, and injury. Physiol Rev. (2019) 99:1047–78. 10.1152/physrev.00020.2018 30648461

[65] Ruan JW Liao YC Lua I Li MH Hsu CY Chen JH. Human pituitary tumor-transforming gene 1 overexpression reinforces oncogene-induced senescence through CXCR2/p21 signaling in breast cancer cells. Breast Cancer Res. (2012) 14:R106. 10.1186/bcr3226 22789011 PMC3680924

[66] Xu H Lin F Wang Z Yang L Meng J Ou Z. CXCR2 promotes breast cancer metastasis and chemoresistance via suppression of AKT1 and activation of COX2. Cancer Lett. (2018) 412:69–80. 10.1016/j.canlet.2017.09.030 28964785

[67] Coppe JP Patil CK Rodier F Sun Y Munoz DP Goldstein J. Senescence-associated secretory phenotypes reveal cell-nonautonomous functions of oncogenic RAS and the p53 tumor suppressor. PLoS Biol. (2008) 6:2853–68. 10.1371/journal.pbio.0060301 19053174 PMC2592359

[68] Ohanna M Giuliano S Bonet C Imbert V Hofman V Zangari J. Senescent cells develop a PARP-1 and nuclear factor-{kappa}B-associated secretome (PNAS). Genes Dev. (2011) 25:1245–61. 10.1101/gad.625811 21646373 PMC3127427

[69] Eyman D Damodarasamy M Plymate SR Reed MJ. CCL5 secreted by senescent aged fibroblasts induces proliferation of prostate epithelial cells and expression of genes that modulate angiogenesis. J Cell Physiol. (2009) 220:376–81. 10.1002/jcp.21776 19360811 PMC2846281

[70] Kim YH Choi YW Lee J Soh EY Kim JH Park TJ. Senescent tumor cells lead the collective invasion in thyroid cancer. Nat Commun. (2017) 8:15208. 10.1038/ncomms15208 28489070 PMC5436223

[71] Eggert T Wolter K Ji J Ma C Yevsa T Klotz S. Distinct functions of senescence-associated immune responses in liver tumor surveillance and tumor progression. Cancer Cell. (2016) 30:533–47. 10.1016/j.ccell.2016.09.003 27728804 PMC7789819

[72] Iannello A Thompson TW Ardolino M Lowe SW Raulet DH. p53-dependent chemokine production by senescent tumor cells supports NKG2D-dependent tumor elimination by natural killer cells. J Exp Med. (2013) 210:2057–69. 10.1084/jem.20130783 24043758 PMC3782044

[73] Prasetyanti PR Medema JP. Intra-tumor heterogeneity from a cancer stem cell perspective. Mol Cancer. (2017) 16:41. 10.1186/s12943-017-0600-4 28209166 PMC5314464

[74] Batlle E Clevers H. Cancer stem cells revisited. Nat Med. (2017) 23:1124–34. 10.1038/nm.4409 28985214

[75] Dittmer J. Breast cancer stem cells: features, key drivers and treatment options. Semin Cancer Biol. (2018) 53:59–74. 10.1016/j.semcancer.2018.07.007 30059727

[76] Charafe-Jauffret E Ginestier C Iovino F Wicinski J Cervera N Finetti P. Breast cancer cell lines contain functional cancer stem cells with metastatic capacity and a distinct molecular signature. Cancer Res. (2009) 69:1302–13. 10.1158/0008-5472.CAN-08-2741 19190339 PMC2819227

[77] Ginestier C Liu S Diebel ME Korkaya H Luo M Brown M. CXCR1 blockade selectively targets human breast cancer stem cells *in vitro* and in xenografts. J Clin Invest. (2010) 120:485–97. 10.1172/JCI39397 20051626 PMC2810075

[78] Wang N Zheng Y Gu J Cai Y Wang S Zhang F. Network-pharmacology-based validation of TAMS/CXCL-1 as key mediator of XIAOPI formula preventing breast cancer development and metastasis. Sci Rep. (2017) 7:14513. 10.1038/s41598-017-15030-3 29109519 PMC5674025

[79] Singh JK Farnie G Bundred NJ Simoes BM Shergill A Landberg G. Targeting CXCR1/2 significantly reduces breast cancer stem cell activity and increases the efficacy of inhibiting HER2 via HER2-dependent and -independent mechanisms. Clin Cancer Res. (2013) 19:643–56. 10.1158/1078-0432.CCR-12-1063 23149820 PMC4868141

[80] Samanta D Gilkes DM Chaturvedi P Xiang L Semenza GL. Hypoxia-inducible factors are required for chemotherapy resistance of breast cancer stem cells. Proc Natl Acad Sci USA. (2014) 111:E5429–38. 10.1073/pnas.1421438111 25453096 PMC4273385

[81] Jia D Li L Andrew S Allan D Li X Lee J. An autocrine inflammatory forward-feedback loop after chemotherapy withdrawal facilitates the repopulation of drug-resistant breast cancer cells. Cell Death Dis. (2017) 8:e2932. 10.1038/cddis.2017.319 28703802 PMC5550865

[82] Tsuyada A Chow A Wu J Somlo G Chu P Loera S. CCL2 mediates cross-talk between cancer cells and stromal fibroblasts that regulates breast cancer stem cells. Cancer Res. (2012) 72:2768–79. 10.1158/0008-5472.CAN-11-3567 22472119 PMC3367125

[83] Jiao X Velasco-Velazquez MA Wang M Li Z Rui H Peck AR. CCR5 governs DNA damage repair and breast cancer stem cell expansion. Cancer Res. (2018) 78:1657–71. 10.1158/0008-5472.CAN-17-0915 29358169 PMC6331183

[84] Kong L Guo S Liu C Zhao Y Feng C Liu Y. Overexpression of SDF-1 activates the NF-kappaB pathway to induce epithelial to mesenchymal transition and cancer stem cell-like phenotypes of breast cancer cells. Int J Oncol. (2016) 48:1085–94. 10.3892/ijo.2016.3343 26782945

[85] Ablett MP O'Brien CS Sims AH Farnie G Clarke RB. A differential role for CXCR4 in the regulation of normal versus malignant breast stem cell activity. Oncotarget. (2014) 5:599–612. 10.18632/oncotarget.1169 24583601 PMC3996659

[86] Huang M Li Y Zhang H Nan F. Breast cancer stromal fibroblasts promote the generation of CD44+CD24- cells through SDF-1/CXCR4 interaction. J Exp Clin Cancer Res. (2010) 29:80. 10.1186/1756-9966-29-80 20569497 PMC2911413

[87] Dubrovska A Hartung A Bouchez LC Walker JR Reddy VA Cho CY. CXCR4 activation maintains a stem cell population in tamoxifen-resistant breast cancer cells through AhR signalling. Br J Cancer. (2012) 107:43–52. 10.1038/bjc.2012.105 22644306 PMC3389396

[88] Wang N Liu W Zheng Y Wang S Yang B Li M. CXCL1 derived from tumor-associated macrophages promotes breast cancer metastasis via activating NF-kappaB/SOX4 signaling. Cell Death Dis. (2018) 9:880. 10.1038/s41419-018-0876-3 30158589 PMC6115425

[89] Fernando RI Castillo MD Litzinger M Hamilton DH Palena C. IL-8 signaling plays a critical role in the epithelial-mesenchymal transition of human carcinoma cells. Cancer Res. (2011) 71:5296–306. 10.1158/0008-5472.CAN-11-0156 21653678 PMC3148346

[90] Wang L Tang C Cao H Li K Pang X Zhong L. Activation of IL-8 via PI3K/Akt-dependent pathway is involved in leptin-mediated epithelial-mesenchymal transition in human breast cancer cells. Cancer Biol Ther. (2015) 16:1220–30. 10.1080/15384047.2015.1056409 26121010 PMC4622725

[91] Valeta-Magara A Gadi A Volta V Walters B Arju R Giashuddin S. Inflammatory breast cancer promotes development of M2 tumor-associated macrophages and cancer mesenchymal cells through a complex chemokine network. Cancer Res. (2019) 79:3360–71. 10.1158/0008-5472.CAN-17-2158 31043378 PMC7331114

[92] Bhat K Sarkissyan M Wu Y Vadgama JV. GROalpha overexpression drives cell migration and invasion in triple negative breast cancer cells. Oncol Rep. (2017) 38:21–30. 10.3892/or.2017.5668 28560447 PMC5492847

[93] Hu Q Myers M Fang W Yao M Brummer G Hawj J. Role of ALDH1A1 and HTRA2 expression in CCL2/CCR2-mediated breast cancer cell growth and invasion. Biol Open. (2019) 8:bio040873. 10.1242/bio.040873 31208996 PMC6679398

[94] Wang LP Cao J Zhang J Wang BY Hu XC Shao ZM. The human chemokine receptor CCRL2 suppresses chemotaxis and invasion by blocking CCL2-induced phosphorylation of p38 MAPK in human breast cancer cells. Med Oncol. (2015) 32:254. 10.1007/s12032-015-0696-6 26487662

[95] Song X Zhou X Qin Y Yang J Wang Y Sun Z. Emodin inhibits epithelialmesenchymal transition and metastasis of triple negative breast cancer via antagonism of CCchemokine ligand 5 secreted from adipocytes. Int J Mol Med. (2018) 42:579–88. 10.3892/ijmm.2018.3638 29693154

[96] Lin S Sun L Lyu X Ai X Du D Su N. Lactate-activated macrophages induced aerobic glycolysis and epithelial-mesenchymal transition in breast cancer by regulation of CCL5-CCR5 axis: a positive metabolic feedback loop. Oncotarget. (2017) 8:110426–43. 10.18632/oncotarget.22786 29299159 PMC5746394

[97] Sobolik T Su YJ Wells S Ayers GD Cook RS Richmond A. CXCR4 drives the metastatic phenotype in breast cancer through induction of CXCR2, and activation of MEK and PI3K pathways. Mol. Biol. Cell. (2014) 25:566–82. 10.1158/1538-7445.TIM2013-A53 24403602 PMC3937084

[98] Shan S Lv Q Zhao Y Liu C Sun Y Xi K. Wnt/β-catenin pathway is required for epithelial to mesenchymal transition in CXCL12 over expressed breast cancer cells. Int J Clin Exp Pathol. (2015) 8:12357–67. 26722422 PMC4680367

[99] Azenshtein E Luboshits G Shina S Neumark E Shahbazian D Weil M. The CC chemokine RANTES in breast carcinoma progression: regulation of expression and potential mechanisms of promalignant activity. Cancer Res. (2002) 62:1093–102. 11861388

[100] Kim JE Kim HS Shin YJ Lee CS Won C Lee SA. LYR71, a derivative of trimeric resveratrol, inhibits tumorigenesis by blocking STAT3-mediated matrix metalloproteinase 9 expression. Exp Mol Med. (2008) 40:514–22. 10.3858/emm.2008.40.5.514 18985009 PMC2679359

[101] Li S Kendall SE Raices R Finlay J Covarrubias M Liu Z. TWIST1 associates with NF- κB subunit RELA via carboxyl-terminal WR domain to promote cell autonomous invasion through IL8 production. BMC Biol. (2012) 10:73. 10.1186/1741-7007-10-73 22891766 PMC3482588

[102] Swamydas M Ricci K Rego SL Dreau D. Mesenchymal stem cell-derived CCL-9 and CCL-5 promote mammary tumor cell invasion and the activation of matrix metalloproteinases. Cell Adh Migr. (2013) 7:315–24. 10.4161/cam.25138 23722213 PMC3711999

[103] Yang C Yu H Chen R Tao K Jian L Peng M. CXCL1 stimulates migration and invasion in ERnegative breast cancer cells via activation of the ERK/MMP2/9 signaling axis. Int J Oncol. (2019) 55:684–96. 10.3892/ijo.2019.4840 31322183 PMC6685590

[104] Mao L Yuan L Slakey LM Jones FE Burow ME Hill SM. Inhibition of breast cancer cell invasion by melatonin is mediated through regulation of the p38 mitogen-activated protein kinase signaling pathway. Breast Cancer Res. (2010) 12:R107. 10.1186/bcr2794 21167057 PMC3046452

[105] Fernandis AZ Prasad A Band H Klosel R Ganju RK. Regulation of CXCR4-mediated chemotaxis and chemoinvasion of breast cancer cells. Oncogene. (2004) 23:157–67. 10.1038/sj.onc.1206910 14712221

[106] Wani N Nasser MW Ahirwar DK Zhao H Miao Z Shilo K. C-X-C motif chemokine 12/C-X-C chemokine receptor type 7 signaling regulates breast cancer growth and metastasis by modulating the tumor microenvironment. Breast Cancer Res. (2014) 16:R54. 10.1186/bcr3665 24886617 PMC4076630

[107] Hernandez L Magalhaes MA Coniglio SJ Condeelis JS Segall JE. Opposing roles of CXCR4 and CXCR7 in breast cancer metastasis. Breast Cancer Res. (2011) 13:R128. 10.1186/bcr3074 22152016 PMC3326570

[108] Najafi M Mortezaee K Ahadi R. Cancer stem cell (a)symmetry & plasticity: tumorigenesis and therapy relevance. Life Sci. (2019) 231:116520. 10.1016/j.lfs.2019.05.076 31158379

[109] Rodriguez D Ramkairsingh M Lin X Kapoor A Major P Tang D. The central contributions of breast cancer stem cells in developing resistance to endocrine therapy in estrogen receptor (ER)-positive breast cancer. Cancers. (2019) 11:1028. 10.3390/cancers11071028 31336602 PMC6678134

[110] Chen DR Lu DY Lin HY Yeh WL. Mesenchymal stem cell-induced doxorubicin resistance in triple negative breast cancer. Biomed Res Int. (2014) 2014:532161. 10.1155/2014/532161 25140317 PMC4124237

[111] Sharma B Nawandar DM Nannuru KC Varney ML Singh RK. Targeting CXCR2 enhances chemotherapeutic response, inhibits mammary tumor growth, angiogenesis, and lung metastasis. Mol Cancer Ther. (2013) 12:799–808. 10.1158/1535-7163.MCT-12-0529 23468530 PMC3653628

[112] Shi Z Yang WM Chen LP Yang DH Zhou Q Zhu J. Enhanced chemosensitization in multidrug-resistant human breast cancer cells by inhibition of IL-6 and IL-8 production. Breast Cancer Res Treat. (2012) 135:737–47. 10.1007/s10549-012-2196-0 22923236

[113] Acharyya S Oskarsson T Vanharanta S Malladi S Kim J Morris PG. A CXCL1 paracrine network links cancer chemoresistance and metastasis. Cell. (2012) 150:165–78. 10.1016/j.cell.2012.04.042 22770218 PMC3528019

[114] Greco SJ Patel SA Bryan M Pliner LF Banerjee D Rameshwar P. AMD3100-mediated production of interleukin-1 from mesenchymal stem cells is key to chemosensitivity of breast cancer cells. Am J Cancer Res. (2011) 1:701–15. 22016821 PMC3195931

[115] Li D Ji H Niu X Yin L Wang Y Gu Y. Tumor-associated macrophages secrete CC-chemokine ligand 2 and induce tamoxifen resistance by activating PI3K/Akt/mTOR in breast cancer. Cancer Sci. (2020) 111:47–58. 10.1111/cas.14230 31710162 PMC6942430

[116] Rhodes LV Short SP Neel NF Salvo VA Zhu Y Elliott S. Cytokine receptor CXCR4 mediates estrogen-independent tumorigenesis, metastasis, and resistance to endocrine therapy in human breast cancer. Cancer Res. (2011) 71:603–13. 10.1158/0008-5472.CAN-10-3185 21123450 PMC3140407

[117] Hao M Weng X Wang Y Sun X Yan T Li Y. Targeting CXCR7 improves the efficacy of breast cancer patients with tamoxifen therapy. Biochem Pharmacol. (2018) 147:128–40. 10.1016/j.bcp.2017.11.013 29175422

[118] Sun L Wang Q Chen B Zhao Y Shen B Wang H. Gastric cancer mesenchymal stem cells derived IL-8 induces PD-L1 expression in gastric cancer cells via STAT3/mTOR-c-Myc signal axis. Cell Death Dis. (2018) 9:928. 10.1038/s41419-018-0988-9 30206229 PMC6134105

[119] Li Z Zhou J Zhang J Li S Wang H Du J. Cancer-associated fibroblasts promote PD-L1 expression in mice cancer cells via secreting CXCL5. Int J Cancer. (2019) 145:1946–57. 10.1002/ijc.32278 30873585 PMC6767568

[120] Liu C Yao Z Wang J Zhang W Yang Y Zhang Y Macrophage-derived CCL5 facilitates immune escape of colorectal cancer cells via the p65/STAT3-CSN5-PD-L1 pathway. Cell Death Differ. (2019). 10.1038/s41418-019-0460-0 PMC7244707 31802034

[121] Lin C He H Liu H Li R Chen Y Qi Y. Tumour-associated macrophages-derived CXCL8 determines immune evasion through autonomous PD-L1 expression in gastric cancer. Gut. (2019) 68:1764–73. 10.1136/gutjnl-2018-316324 30661053

[122] Zhu H Gu Y Xue Y Yuan M Cao X Liu Q. CXCR2(+) MDSCs promote breast cancer progression by inducing EMT and activated T cell exhaustion. Oncotarget. (2017) 8:114554–67. 10.18632/oncotarget.23020 29383101 PMC5777713

[123] Highfill SL Cui Y Giles AJ Smith JP Zhang H Morse E. Disruption of CXCR2-mediated MDSC tumor trafficking enhances anti-PD1 efficacy. Sci Transl Med. (2014) 6:237ra67. 10.1126/scitranslmed.3007974 24848257 PMC6980372

[124] Sai B Dai Y Fan S Wang F Wang L Li Z. Cancer-educated mesenchymal stem cells promote the survival of cancer cells at primary and distant metastatic sites via the expansion of bone marrow-derived-PMN-MDSCs. Cell Death Dis. (2019) 10:941. 10.1038/s41419-019-2149-1 31819035 PMC6901580

[125] Flores-Toro JA Luo D Gopinath A Sarkisian MR Campbell JJ Charo IF. CCR2 inhibition reduces tumor myeloid cells and unmasks a checkpoint inhibitor effect to slow progression of resistant murine gliomas. Proc Natl Acad Sci USA. (2020) 117:1129–38. 10.1073/pnas.1910856117 31879345 PMC6969504

[126] Zhang S Zhong M Wang C Xu Y Gao WQ Zhang Y. CCL5-deficiency enhances intratumoral infiltration of CD8(+) T cells in colorectal cancer. Cell Death Dis. (2018) 9:766. 10.1038/s41419-018-0796-2 29991744 PMC6039518

[127] Jung HBA Ebsworth K Ertl L Schall T Charo I Combination therapy of chemokine receptor inhibition plus PDL-1 blockade potentiates anti-tumor effects in a murine model of breast cancer. J Immuno Ther Cancer. (2015) 3:P227 10.1186/2051-1426-3-S2-P227

[128] Chen IX Chauhan VP Posada J Ng MR Wu MW Adstamongkonkul P. Blocking CXCR4 alleviates desmoplasia, increases T-lymphocyte infiltration, and improves immunotherapy in metastatic breast cancer. Proc Natl Acad Sci USA. (2019) 116:4558–66. 10.1073/pnas.1815515116 30700545 PMC6410779

[129] Zeng Y Li B Liang Y Reeves PM Qu X Ran C. Dual blockade of CXCL12-CXCR4 and PD-1-PD-L1 pathways prolongs survival of ovarian tumor-bearing mice by prevention of immunosuppression in the tumor microenvironment. FASEB J. (2019) 33:6596–608. 10.1096/fj.201802067RR 30802149 PMC6463916

[130] Feig C Jones JO Kraman M Wells RJ Deonarine A Chan DS. Targeting CXCL12 from FAP-expressing carcinoma-associated fibroblasts synergizes with anti-PD-L1 immunotherapy in pancreatic cancer. Proc Natl Acad Sci USA. (2013) 110:20212–7. 10.1073/pnas.1320318110 24277834 PMC3864274

[131] Lee CH Kakinuma T Wang J Zhang H Palmer DC Restifo NP. Sensitization of B16 tumor cells with a CXCR4 antagonist increases the efficacy of immunotherapy for established lung metastases. Mol Cancer Ther. (2006) 5:2592–9. 10.1158/1535-7163.MCT-06-0310 17041104 PMC2228334

[132] Zboralski D Hoehlig K Eulberg D Fromming A Vater A. Increasing tumor-infiltrating T cells through inhibition of CXCL12 with NOX-A12 synergizes with PD-1 blockade. Cancer Immunol Res. (2017) 5:950–6. 10.1158/2326-6066.CIR-16-0303 28963140

[133] D'Alterio C Buoncervello M Ierano C Napolitano M Portella L Rea G. Targeting CXCR4 potentiates anti-PD-1 efficacy modifying the tumor microenvironment and inhibiting neoplastic PD-1. J Exp Clin Cancer Res. (2019) 38:432. 10.1186/s13046-019-1420-8 31661001 PMC6819555

[134] Dangaj D Bruand M Grimm AJ Ronet C Barras D Duttagupta PA. Cooperation between constitutive and inducible chemokines enables T Cell engraftment and immune attack in solid tumors. Cancer Cell. (2019) 35:885–900 e10. 10.1016/j.ccell.2019.05.004 31185212 PMC6961655

[135] Walser TC Rifat S Ma X Kundu N Ward C Goloubeva O Antagonism of CXCR3 inhibits lung metastasis in a murine model of metastatic breast cancer. Cancer Res. (2006) 66:7701–7. 10.1158/0008-5472.CAN-06-0709 16885372

[136] Goldberg-Bittman L Sagi-Assif O Meshel T Nevo I Levy-Nissenbaum O Yron I. Cellular characteristics of neuroblastoma cells: regulation by the ELR–CXC chemokine CXCL10 and expression of a CXCR3-like receptor. Cytokine. (2005) 29:105–17. 10.1016/j.cyto.2004.10.003 15613278

[137] Ben-Baruch A (2008). Expert Commentary: The Chemokine Receptor CXCR3 and its Ligands in Malignancy: Do they Act as Double-Edged Swords? New York, NY: Chemokine research trendSpringer Publishers.

[138] Tokunaga R Zhang W Naseem M Puccini A Berger MD Soni S. CXCL9, CXCL10, CXCL11/CXCR3 axis for immune activation - a target for novel cancer therapy. Cancer Treat Rev. (2018) 63:40–7. 10.1016/j.ctrv.2017.11.007 29207310 PMC5801162

[139] Vilgelm AE Richmond A. Chemokines modulate immune surveillance in tumorigenesis, metastasis, and response to immunotherapy. Front Immunol. (2019) 10:333. 10.3389/fimmu.2019.00333 30873179 PMC6400988

[140] Weilbaecher KN Guise TA McCauley LK. Cancer to bone: a fatal attraction. Nat Rev Cancer. (2011) 11:411–25. 10.1038/nrc3055 21593787 PMC3666847

[141] Brylka LJ Schinke T. Chemokines in physiological and pathological bone remodeling. Front Immunol. (2019) 10:2182. 10.3389/fimmu.2019.02182 31572390 PMC6753917

[142] Koizumi K Saitoh Y Minami T Takeno N Tsuneyama K Miyahara T. Role of CX3CL1/fractalkine in osteoclast differentiation and bone resorption. J Immunol. (2009) 183:7825–31. 10.4049/jimmunol.0803627 19923448

[143] Hoshino A Ueha S Hanada S Imai T Ito M Yamamoto K. Roles of chemokine receptor CX3CR1 in maintaining murine bone homeostasis through the regulation of both osteoblasts and osteoclasts. J Cell Sci. (2013) 126:1032–45. 10.1242/jcs.113910 23264747

[144] Coniglio SJ. Role of tumor-derived chemokines in osteolytic bone metastasis. Front Endocrinol. (2018) 9:313. 10.3389/fendo.2018.00313 29930538 PMC5999726

[145] Kelly T Suva LJ Nicks KM MacLeod V Sanderson RD. Tumor-derived syndecan-1 mediates distal cross-talk with bone that enhances osteoclastogenesis. J Bone Miner Res. (2010) 25:1295–304. 10.1002/jbmr.16 20200931 PMC3148092

[146] Kamalakar A Bendre MS Washam CL Fowler TW Carver A Dilley JD. Circulating interleukin-8 levels explain breast cancer osteolysis in mice and humans. Bone. (2014) 61:176–85. 10.1016/j.bone.2014.01.015 24486955 PMC3967592

[147] Yang YH Buhamrah A Schneider A Lin YL Zhou H Bugshan A. Semaphorin 4D promotes skeletal metastasis in breast cancer. PLoS ONE. (2016) 11:e0150151. 10.1371/journal.pone.0150151 26910109 PMC4766104

[148] Bendre MS Margulies AG Walser B Akel NS Bhattacharrya S Skinner RA. Tumor-derived interleukin-8 stimulates osteolysis independent of the receptor activator of nuclear factor-kappaB ligand pathway. Cancer Res. (2005) 65:11001–9. 10.1158/0008-5472.CAN-05-2630 16322249

[149] Pathi SP Kowalczewski C Tadipatri R Fischbach C. A novel 3-D mineralized tumor model to study breast cancer bone metastasis. PLoS ONE. (2010) 5:e8849. 10.1371/journal.pone.0008849 20107512 PMC2809751

[150] Bussard KM Okita N Sharkey N Neuberger T Webb A Mastro AM. Localization of osteoblast inflammatory cytokines MCP-1 and VEGF to the matrix of the trabecula of the femur, a target area for metastatic breast cancer cell colonization. Clin Exp Metastasis. (2010) 27:331–40. 10.1007/s10585-010-9330-3 20446021

[151] Lu X Kang Y. Chemokine (C-C motif) ligand 2 engages CCR2+ stromal cells of monocytic origin to promote breast cancer metastasis to lung and bone. J Biol Chem. (2009) 284:29087–96. 10.1074/jbc.M109.035899 19720836 PMC2781454

[152] He Z He J Liu Z Xu J Yi SF Liu H. MAPK11 in breast cancer cells enhances osteoclastogenesis and bone resorption. Biochimie. (2014) 106:24–32. 10.1016/j.biochi.2014.07.017 25066918 PMC4250302

[153] Sasaki S Baba T Nishimura T Hayakawa Y Hashimoto S Gotoh N. Essential roles of the interaction between cancer cell-derived chemokine, CCL4, and intra-bone CCR5-expressing fibroblasts in breast cancer bone metastasis. Cancer Lett. (2016) 378:23–32. 10.1016/j.canlet.2016.05.005 27177471

[154] Bussard KM Mutkus L Stumpf K Gomez-Manzano C Marini FC. Tumor-associated stromal cells as key contributors to the tumor microenvironment. Breast Cancer Res. (2016) 18:84. 10.1186/s13058-016-0740-2 27515302 PMC4982339

[155] Liao Z Tan ZW Zhu P Tan NS. Cancer-associated fibroblasts in tumor microenvironment - accomplices in tumor malignancy. Cell Immunol. (2019) 343:103729. 10.1016/j.cellimm.2017.12.003 29397066

[156] Buchsbaum RJ Oh SY. Breast cancer-associated fibroblasts: where we are and where we need to go. Cancers. (2016) 8:19. 10.3390/cancers8020019 26828520 PMC4773742

[157] LeBleu VS Kalluri R. A peek into cancer-associated fibroblasts: origins, functions and translational impact. Dis Model Mech. (2018) 11:dmm029447. 10.1242/dmm.029447 29686035 PMC5963854

[158] Hill BS Pelagalli A Passaro N Zannetti A. Tumor-educated mesenchymal stem cells promote pro-metastatic phenotype. Oncotarget. (2017) 8:73296–311. 10.18632/oncotarget.20265 29069870 PMC5641213

[159] Liubomirski Y Lerrer S Meshel T Rubinstein-Achiasaf L Morein D Wiemann S. Tumor-stroma-inflammation networks promote pro-metastatic chemokines and aggressiveness characteristics in triple-negative breast cancer. Front Immunol. (2019) 10:757. 10.3389/fimmu.2019.00757 31031757 PMC6473166

[160] Liubomirski Y Lerrer S Meshel T Morein D Rubinstein-Achiasaf L Sprinzak D. Notch-mediated tumor-stroma-inflammation networks promote invasive properties and CXCL8 expression in triple-negative breast cancer. Front Immunol. (2019) 10:804. 10.3389/fimmu.2019.00804 31105691 PMC6492532

[161] Potter SM Dwyer RM Hartmann MC Khan S Boyle MP Curran CE. Influence of stromal-epithelial interactions on breast cancer *in vitro* and *in vivo*. Breast Cancer Res Treat. (2012) 131:401–11. 10.1007/s10549-011-1410-9 21344235

[162] Hembruff SL Jokar I Yang L Cheng N. Loss of transforming growth factor-beta signaling in mammary fibroblasts enhances CCL2 secretion to promote mammary tumor progression through macrophage-dependent and -independent mechanisms. Neoplasia. (2010) 12:425–33. 10.1593/neo.10200 20454514 PMC2864480

[163] Mi Z Bhattacharya SD Kim VM Guo H Talbot LJ Kuo PC. Osteopontin promotes CCL5-mesenchymal stromal cell-mediated breast cancer metastasis. Carcinogenesis. (2011) 32:477–87. 10.1093/carcin/bgr009 21252118 PMC3105582

[164] Karnoub AE Dash AB Vo AP Sullivan A Brooks MW Bell GW. Mesenchymal stem cells within tumour stroma promote breast cancer metastasis. Nature. (2007) 449:557–63. 10.1038/nature06188 17914389

[165] Kalluri R The biology and function of fibroblasts in cancer. Nat Rev Cancer. (2016) 16:582–98. 10.1038/nrc.2016.73 27550820

[166] Quante M Tu SP Tomita H Gonda T Wang SS Takashi S. Bone marrow-derived myofibroblasts contribute to the mesenchymal stem cell niche and promote tumor growth. Cancer Cell. (2011) 19:257–72. 10.1016/j.ccr.2011.01.020 21316604 PMC3060401

[167] Jotzu C Alt E Welte G Li J Hennessy BT Devarajan E. Adipose tissue derived stem cells differentiate into carcinoma-associated fibroblast-like cells under the influence of tumor derived factors. Cell Oncol. (2011) 34:55–67. 10.1007/s13402-011-0012-1 21327615 PMC13014585

[168] Shangguan L Ti X Krause U Hai B Zhao Y Yang Z. Inhibition of TGF-beta/Smad signaling by BAMBI blocks differentiation of human mesenchymal stem cells to carcinoma-associated fibroblasts and abolishes their protumor effects. Stem Cells. (2012) 30:2810–9. 10.1002/stem.1251 23034983

[169] Mishra P Banerjee D Ben-Baruch A. Chemokines at the crossroads of tumor-fibroblast interactions that promote malignancy. J Leukoc Biol. (2011) 89:31–9. 10.1189/jlb.0310182 20628066

[170] Dwyer RM Potter-Beirne SM Harrington KA Lowery AJ Hennessy E Murphy JM. Monocyte chemotactic protein-1 secreted by primary breast tumors stimulates migration of mesenchymal stem cells. Clin Cancer Res. (2007) 13:5020–7. 10.1158/1078-0432.CCR-07-0731 17785552

[171] Klopp AH Spaeth EL Dembinski JL Woodward WA Munshi A Meyn RE. Tumor irradiation increases the recruitment of circulating mesenchymal stem cells into the tumor microenvironment. Cancer Res. (2007) 67:11687–95. 10.1158/0008-5472.CAN-07-1406 18089798 PMC4329784

[172] Sharon Y Raz Y Cohen N Ben-Shmuel A Schwartz H Geiger T. Tumor-derived osteopontin reprograms normal mammary fibroblasts to promote inflammation and tumor growth in breast cancer. Cancer Res. (2015) 75:963–73. 10.1158/0008-5472.CAN-14-1990 25600648

[173] Sun X Glynn DJ Hodson LJ Huo C Britt K Thompson EW. CCL2-driven inflammation increases mammary gland stromal density and cancer susceptibility in a transgenic mouse model. Breast Cancer Res. (2017) 19:4. 10.1186/s13058-016-0796-z 28077158 PMC5225654

[174] Chaturvedi P Gilkes DM Takano N Semenza GL. Hypoxia-inducible factor-dependent signaling between triple-negative breast cancer cells and mesenchymal stem cells promotes macrophage recruitment. Proc Natl Acad Sci USA. (2014) 111:E2120–E2129. 10.1073/pnas.1406655111 24799675 PMC4034192

[175] Gallo M De Luca A Lamura L Normanno N. Zoledronic acid blocks the interaction between mesenchymal stem cells and breast cancer cells: implications for adjuvant therapy of breast cancer. Ann. Oncol. (2011) 23:597–604. 10.1158/1538-7445.AM10-565 21551002

[176] Orimo A Gupta PB Sgroi DC Arenzana-Seisdedos F Delaunay T Naeem R. Stromal fibroblasts present in invasive human breast carcinomas promote tumor growth and angiogenesis through elevated SDF-1/CXCL12 secretion. Cell. (2005) 121:335–48. 10.1016/j.cell.2005.02.034 15882617

[177] Kojima Y Acar A Eaton EN Mellody KT Scheel C Ben-Porath I. Autocrine TGF-beta and stromal cell-derived factor-1 (SDF-1) signaling drives the evolution of tumor-promoting mammary stromal myofibroblasts. Proc Natl Acad Sci USA. (2010) 107:20009–14. 10.1073/pnas.1013805107 21041659 PMC2993333

[178] Peng Q Zhao L Hou Y Sun Y Wang L Luo H. Biological characteristics and genetic heterogeneity between carcinoma-associated fibroblasts and their paired normal fibroblasts in human breast cancer. PLoS ONE. (2013) 8:e60321. 10.1371/journal.pone.0060321 23577100 PMC3618271

[179] Rhodes LV Antoon JW Muir SE Elliott S Beckman BS Burow ME. Effects of human mesenchymal stem cells on ER-positive human breast carcinoma cells mediated through ER-SDF-1/CXCR4 crosstalk. Mol Cancer. (2010) 9:295. 10.1186/1476-4598-9-295 21087507 PMC2998478

[180] Al-Ansari MM Hendrayani SF Shehata AI Aboussekhra A. p16(INK4A) represses the paracrine tumor-promoting effects of breast stromal fibroblasts. Oncogene. (2013) 32:2356–64. 10.1038/onc.2012.270 22751126 PMC3679618

[181] Al-Toub M Almohawes M Vishnubalaji R Alfayez M Aldahmash A Kassem M. CXCR7 signaling promotes breast cancer survival in response to mesenchymal stromal stem cell-derived factors. Cell Death Discov. (2019) 5:87. 10.1038/s41420-019-0169-3 30993013 PMC6459874

[182] Yu PF Huang Y Xu CL Lin LY Han YY Sun WH. Downregulation of CXCL12 in mesenchymal stromal cells by TGFbeta promotes breast cancer metastasis. Oncogene. (2017) 36:840–9. 10.1038/onc.2016.252 27669436 PMC5311419

[183] Zhao K Yao Y Luo X Lin B Huang Y Zhou Y. LYG-202 inhibits activation of endothelial cells and angiogenesis through CXCL12/CXCR7 pathway in breast cancer. Carcinogenesis. (2018) 39:588–600. 10.1093/carcin/bgy007 29390073

[184] Miao Z Luker KE Summers BC Berahovich R Bhojani MS Rehemtulla A. CXCR7 (RDC1) promotes breast and lung tumor growth *in vivo* and is expressed on tumor-associated vasculature. Proc Natl Acad Sci USA. (2007) 104:15735–40. 10.1073/pnas.0610444104 17898181 PMC1994579

[185] Qian T Liu Y Dong Y Zhang L Dong Y Sun Y. CXCR7 regulates breast tumor metastasis and angiogenesis *in vivo* and *in vitro*. Mol Med Rep. (2018) 17:3633–9. 10.3892/mmr.2017.8286 29257351 PMC5802168

[186] Torisawa YS Mosadegh B Bersano-Begey T Steele JM Luker KE Luker GD. Microfluidic platform for chemotaxis in gradients formed by CXCL12 source-sink cells. Integr Biol. (2010) 2:680–6. 10.1039/c0ib00041h 20871938 PMC4128891

[187] Inaguma S Riku M Ito H Tsunoda T Ikeda H Kasai K. GLI1 orchestrates CXCR4/CXCR7 signaling to enhance migration and metastasis of breast cancer cells. Oncotarget. (2015) 6:33648–57. 10.18632/oncotarget.5203 26413813 PMC4741792

[188] Zeng XH Ou ZL Yu KD Feng LY Yin WJ Li J. Coexpression of atypical chemokine binders (ACBs) in breast cancer predicts better outcomes. Breast Cancer Res Treat. (2011) 125:715–27. 10.1007/s10549-010-0875-2 20369284

[189] Yu KD Wang X Yang C Zeng XH Shao ZM. Host genotype and tumor phenotype of chemokine decoy receptors integrally affect breast cancer relapse. Oncotarget. (2015) 6:26519–27. 10.18632/oncotarget.4470 26314842 PMC4694919

[190] Jenkins BD Martini RN Hire R Brown A Bennett B Brown I. Atypical chemokine receptor 1 (DARC/ACKR1) in breast tumors is associated with survival, circulating chemokines, tumor-infiltrating immune cells, and african ancestry. Cancer Epidemiol Biomark Prev. (2019) 28:690–700. 10.1158/1055-9965.EPI-18-0955 30944146 PMC6450416

[191] Hou T Liang D Xu L Huang X Huang Y Zhang Y. Atypical chemokine receptors predict lymph node metastasis and prognosis in patients with cervical squamous cell cancer. Gynecol Oncol. (2013) 130:181–7. 10.1016/j.ygyno.2013.04.015 23603371

[192] Maeda S Kuboki S Nojima H Shimizu H Yoshitomi H Furukawa K. Duffy antigen receptor for chemokines (DARC) expressing in cancer cells inhibits tumor progression by suppressing CXCR2 signaling in human pancreatic ductal adenocarcinoma. Cytokine. (2017) 95:12–21. 10.1016/j.cyto.2017.02.007 28214673

[193] Yang C Yu KD Xu WH Chen AX Fan L Ou ZL. Effect of genetic variants in two chemokine decoy receptor genes, DARC and CCBP2, on metastatic potential of breast cancer. PLoS ONE. (2013) 8:e78901. 10.1371/journal.pone.0078901 24260134 PMC3829817

[194] Zhu Q Jiang L Wang X. The expression of Duffy antigen receptor for chemokines by epithelial ovarian cancer decreases growth potential. Oncol Lett. (2017) 13:4302–6. 10.3892/ol.2017.5954 28599431 PMC5452942

[195] Bandyopadhyay S Zhan R Chaudhuri A Watabe M Pai SK Hirota S. Interaction of KAI1 on tumor cells with DARC on vascular endothelium leads to metastasis suppression. Nat Med. (2006) 12:933–8. 10.1038/nm1444 16862154

[196] Wang J Ou ZL Hou YF Luo JM Shen ZZ Ding J. Enhanced expression of Duffy antigen receptor for chemokines by breast cancer cells attenuates growth and metastasis potential. Oncogene. (2006) 25:7201–11. 10.1038/sj.onc.1209703 16785997

[197] Khanna P Chung CY Neves RI Robertson GP Dong C. CD82/KAI expression prevents IL-8-mediated endothelial gap formation in late-stage melanomas. Oncogene. (2014) 33:2898–908. 10.1038/onc.2013.249 23873025

[198] Wu FY Ou ZL Feng LY Luo JM Wang LP Shen ZZ. Chemokine decoy receptor d6 plays a negative role in human breast cancer. Mol Cancer Res. (2008) 6:1276–88. 10.1158/1541-7786.MCR-07-2108 18708360

[199] Wu FY Fan J Tang L Zhao YM Zhou CC. Atypical chemokine receptor D6 inhibits human non-small cell lung cancer growth by sequestration of chemokines. Oncol Lett. (2013) 6:91–5. 10.3892/ol.2013.1358 23946783 PMC3742824

[200] Nibbs RJ Gilchrist DS King V Ferra A Forrow S Hunter KD. The atypical chemokine receptor D6 suppresses the development of chemically induced skin tumors. J Clin Invest. (2007) 117:1884–92. 10.1172/JCI30068 17607362 PMC1904306

[201] Vetrano S Borroni EM Sarukhan A Savino B Bonecchi R Correale C. The lymphatic system controls intestinal inflammation and inflammation-associated colon cancer through the chemokine decoy receptor D6. Gut. (2010) 59:197–206. 10.1136/gut.2009.183772 19846409

[202] Savino B Caronni N Anselmo A Pasqualini F Borroni EM Basso G. ERK-dependent downregulation of the atypical chemokine receptor D6 drives tumor aggressiveness in Kaposi sarcoma. Cancer Immunol Res. (2014) 2:679–89. 10.1158/2326-6066.CIR-13-0202 24844911

[203] Hansell CAH Fraser AR Hayes AJ Pingen M Burt CL Lee KM. The Atypical chemokine receptor ackr2 constrains NK cell migratory activity and promotes metastasis. J Immunol. (2018) 201:2510–9. 10.4049/jimmunol.1800131 30158126 PMC6176105

[204] Massara M Bonavita O Savino B Caronni N Mollica Poeta V Sironi M. ACKR2 in hematopoietic precursors as a checkpoint of neutrophil release and anti-metastatic activity. Nat Commun. (2018) 9:676. 10.1038/s41467-018-03080-8 29445158 PMC5813042

[205] Sjoberg E Meyrath M Milde L Herrera M Lovrot J Hagerstrand D. A novel ACKR2-dependent role of fibroblast-derived CXCL14 in epithelial-to-mesenchymal transition and metastasis of breast cancer. Clin Cancer Res. (2019) 25:3702–17. 10.1158/1078-0432.CCR-18-1294 30850359

[206] Zhu Y Tang W Liu Y Wang G Liang Z Cui L. CCX-CKR expression in colorectal cancer and patient survival. Int J Biol Markers. (2014) 29:e40–e48. 10.5301/jbm.5000057 24338720

[207] Shi JY Yang LX Wang ZC Wang LY Zhou J Wang XY. CC chemokine receptor-like 1 functions as a tumour suppressor by impairing CCR7-related chemotaxis in hepatocellular carcinoma. J Pathol. (2015) 235:546–58. 10.1002/path.4450 25255875

[208] Feng LY Ou ZL Wu FY Shen ZZ Shao ZM. Involvement of a novel chemokine decoy receptor CCX-CKR in breast cancer growth, metastasis and patient survival. Clin Cancer Res. (2009) 15:2962–70. 10.1158/1078-0432.CCR-08-2495 19383822

[209] Ju Y Sun C Wang X. Loss of atypical chemokine receptor 4 facilitates C-C motif chemokine ligand 21-mediated tumor growth and invasion in nasopharyngeal carcinoma. Exp Ther Med. (2019) 17:613–20. 10.3892/etm.2018.7007 30651842 PMC6307432

[210] Harata-Lee Y Turvey ME Brazzatti JA Gregor CE Brown MP Smyth MJ. The atypical chemokine receptor CCX-CKR regulates metastasis of mammary carcinoma via an effect on EMT. Immunol Cell Biol. (2014) 92:815–24. 10.1038/icb.2014.58 25027038

[211] Gao D Fish EN. Chemokines in breast cancer: regulating metabolism. Cytokine. (2018) 109:57–64. 10.1016/j.cyto.2018.02.010 29903574

[212] Gao D Cazares LH Fish EN. CCL5-CCR5 interactions modulate metabolic events during tumor onset to promote tumorigenesis. BMC Cancer. (2017) 17:834. 10.1186/s12885-017-3817-0 29216863 PMC5721608

[213] Price TT Burness ML Sivan A Warner M J Cheng R Lee CH. Dormant breast cancer micrometastases reside in specific bone marrow niches that regulate their transit to and from bone. Sci Transl Med. (2016) 8:340ra73. 10.1126/scitranslmed.aad4059 27225183 PMC8722465

[214] Ruffini PA. The CXCL8-CXCR1/2 axis as a therapeutic target in breast cancer stem-like cells. Front Oncol. (2019) 9:40. 10.3389/fonc.2019.00040 30788286 PMC6373429

[215] Halama N Zoernig I Berthel A Kahlert C Klupp F Suarez-Carmona M. Tumoral immune cell exploitation in colorectal cancer metastases can be targeted effectively by anti-CCR5 therapy in cancer patients. Cancer Cell. (2016) 29:587–601. 10.1016/j.ccell.2016.03.005 27070705

[216] Liu T Li X You S Bhuyan SS Dong L. Effectiveness of AMD3100 in treatment of leukemia and solid tumors: from original discovery to use in current clinical practice. Exp Hematol Oncol. (2015) 5:19. 10.1186/s40164-016-0050-5 27429863 PMC4947283

[217] Weitzenfeld P Ben-Baruch A. The chemokine system, and its CCR5 and CXCR4 receptors, as potential targets for personalized therapy in cancer. Cancer Lett. (2014) 352:36–53. 10.1016/j.canlet.2013.10.006 24141062

[218] Vela M Aris M Llorente M Garcia-Sanz JA Kremer L. Chemokine receptor-specific antibodies in cancer immunotherapy: achievements and challenges. Front Immunol. (2015) 6:12. 10.3389/fimmu.2015.00012 25688243 PMC4311683

[219] Scala S. Molecular pathways: targeting the CXCR4-CXCL12 Axis–untapped potential in the tumor microenvironment. Clin Cancer Res. (2015) 21:4278–85. 10.1158/1078-0432.CCR-14-0914 26199389

